# Electrophysiological Properties of Proprioception-Related Neurons in the Intermediate Thoracolumbar Spinal Cord

**DOI:** 10.1523/ENEURO.0331-23.2024

**Published:** 2024-04-23

**Authors:** Felipe Espinosa, Iliodora V. Pop, Helen C. Lai

**Affiliations:** Department of Neuroscience, UT Southwestern Medical Center, Dallas, Texas 75390

**Keywords:** Clarke's column, patch clamp, proprioception, spinal cord

## Abstract

Proprioception, the sense of limb and body position, is required to produce accurate and precise movements. Proprioceptive sensory neurons transmit muscle length and tension information to the spinal cord. The function of excitatory neurons in the intermediate spinal cord, which receive this proprioceptive information, remains poorly understood. Using genetic labeling strategies and patch-clamp techniques in acute spinal cord preparations in mice, we set out to uncover how two sets of spinal neurons, Clarke's column (CC) and *Atoh1*-lineage neurons, respond to electrical activity and how their inputs are organized. Both sets of neurons are located in close proximity in laminae V–VII of the thoracolumbar spinal cord and have been described to receive proprioceptive signals. We find that a majority of CC neurons have a tonic-firing type and express a distinctive hyperpolarization-activated current (I_h_). *Atoh1*-lineage neurons, which cluster into two spatially distinct populations, are mostly a fading-firing type and display similar electrophysiological properties to each other, possibly due to their common developmental lineage. Finally, we find that CC neurons respond to stimulation of lumbar dorsal roots, consistent with prior knowledge that CC neurons receive hindlimb proprioceptive information. In contrast, using a combination of electrical stimulation, optogenetic stimulation, and transsynaptic rabies virus tracing, we find that *Atoh1*-lineage neurons receive heterogeneous, predominantly local thoracic inputs that include parvalbumin-lineage sensory afferents and local interneuron presynaptic inputs. Altogether, we find that CC and *Atoh1*-lineage neurons have distinct membrane properties and sensory input organization, representing different subcircuit modes of proprioceptive information processing.

## Significance Statement

How excitatory spinal cord neurons in the intermediate spinal cord integrate and relay proprioceptive sensory information is not well understood. Our investigation focuses on two sets of spinal neurons that receive proprioceptive information, but whose electrophysiological response properties have not been previously described. We characterize both their passive and active electrophysiological properties in addition to their input connectivity. We identify unique electrophysiological signatures of each population as well as features of their input organization. We find that a hyperpolarization-activated current distinguishes Clarke's column neurons and that *Atoh1*-lineage neurons receive predominantly local inputs. These experiments lay the foundation for future endeavors aimed at understanding the mechanisms by which proprioceptive information is integrated and relayed through these neurons.

## Introduction

In our daily lives, we move seamlessly through our environment. Whether reaching for a cup of coffee or catching a ball, we have an unconscious sense of the spatial location of each of our body parts. This “sixth sense,” called proprioception, originates from proprioceptive sensory neurons that detect changes in muscle length and tension (Sherrington, 1906; Bosco and Poppele, 2001; Tuthill and Azim, 2018). Alterations in proprioceptive function result in severe locomotor deficits indicating how critical the sense of body position is to motor function (Abelew et al., 2000; Akay et al., 2014; Chesler et al., 2016). How proprioceptive information is received by specific excitatory neurons in the intermediate spinal cord is not well understood.

Most of the research on spinal excitatory proprioceptive circuits has focused on a prominent set of large neurons in the medial part of the thoracic to upper lumbar spinal cord called Clarke's column (CC). CC neurons receive hindlimb proprioceptive information and relay this information to the cerebellum through the ipsilaterally projecting dorsal spinocerebellar tract (DSCT; Oscarsson, 1965; Bosco and Poppele, 2001; Hantman and Jessell, 2010). Recordings of DSCT neurons in anesthetized or spinal cats using either metal electrodes or glass sharp electrodes found that DSCT neurons responded to hindlimb proprioceptive stimuli (Eccles et al., 1961; Oscarsson, 1965; Mann, 1971; Kuno et al., 1973; Edgley and Jankowska, 1988; Bosco and Poppele, 2001). However, recordings with sharp and metal electrodes are limited in their ability to assess the cellular response properties of CC neurons (Matsushita and Hosoya, 1979; Baek et al., 2019; Pop et al., 2022). Therefore, using current genetic and electrophysiological techniques, we aimed to precisely identify CC neurons and record their physiological currents.

**Figure 1.**
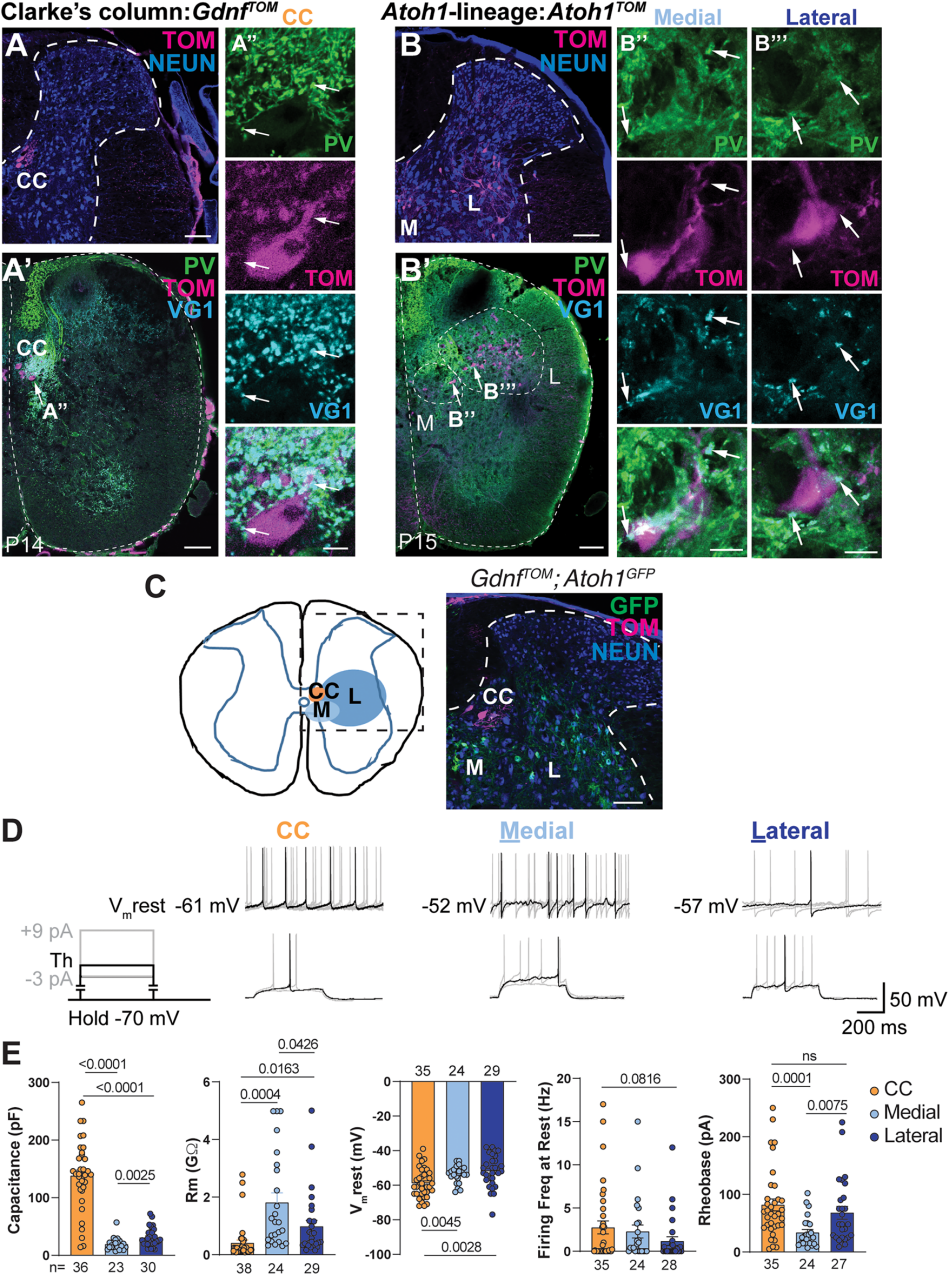
Electrophysiological properties of CC and *Atoh1*-lineage medial and lateral neurons. ***A–B’’’***, Proprioceptive afferent terminals are close to the soma of Clarke's column (CC) and *Atoh1*-lineage medial (M) and lateral (L) neurons. ***A***, Representative lower thoracic image of CC neurons (*Gdnf^TOM^*). ***A’,A’’***, Proprioceptive afferents (PV^+^VG1^+^, arrows) are in close apposition to the soma of CC neurons (TOM). ***B***, Representative lower thoracic image of M and L neurons in *Atoh1^TOM^* mice. ***B’–B’’’***, Proprioceptive afferents (PV^+^VG1^+^, arrows) are in close apposition to the soma of *Atoh1*-lineage M (***B’’***) and L cells (***B’’’***). ***C***, CC and *Atoh1*-lineage neurons (*Gdnf^TOM^; Atoh1^GFP^*) cluster into distinct populations within laminae V–VII of the intermediate spinal cord. ***D***, Top Row. Some cells fire action potentials (APs) at their resting membrane potential V_m_rest (i.e., no holding currents). V_m_rest is shown to the left of each sample trace. Each sample trace contains five traces corresponding to five 2 s sweeps. Only one trace is highlighted in black, and the rest are shown as background in light gray. Bottom row, The protocol schematic to the left indicates that cells were held at −70 mV. After an initial estimation of the rheobase, a more precise determination was done by current steps of 1–3 pA where the first step was a few pAs below the initially estimated threshold. The schematic and traces show the sweep at the threshold in black and sweeps 3 pA below and 9 pA above the threshold in light gray. ***E***, Quantification for capacitance, input resistance (Rm), V_m_rest, firing frequency at rest, and rheobase. PV, parvalbumin; VG1, VGLUT1; TOM, tdTomato. Scale bars: 100 µm (***A, A’***, ***B, B’***, ***C***), 10 µm (***A’’***, ***B’’***, ***B’’’***).

In addition to CC neurons, we focused on defining the electrophysiological properties of a subset of excitatory neurons derived from progenitor cells that express the transcription factor, *Atonal homolog 1* (*Atoh1*; Yuengert et al., 2015; Pop et al., 2022). CC and *Atoh1*-lineage neurons reside in laminae V–VII of the thoracolumbar spinal cord (Fig. 1*A,B*). However, in contrast to the long-range cerebellar-projecting CC neurons, thoracolumbar *Atoh1*-lineage neurons project locally within the spinal cord including synapsing on motor neurons (Pop et al., 2022). Therefore, *Atoh1*-lineage neurons are well-integrated into local spinal circuits. In addition, *Atoh1*-lineage neurons are a heterogeneous group. *Atoh1*-lineage neurons cluster into those near the central canal underneath CC neurons (*Atoh1* medial cells) and lateral to CC neurons (*Atoh1* lateral cells) and are a mixture of contralaterally and ipsilaterally projecting neurons (Bermingham et al., 2001; Wilson et al., 2008; Kaneyama and Shirasaki, 2018; Pop et al., 2022). Given the spatial heterogeneity of *Atoh1*-lineage neurons, we set out to determine if *Atoh1*-lineage medial and lateral neurons had distinguishing electrophysiological signatures.

Finally, while CC neurons are well-established to receive hindlimb proprioceptive inputs, the input organization to *Atoh1*-lineage neurons is less clear. Proprioceptive sensory neuron presynaptic terminals [parvalbumin (PV^+^) and Vesicular glutamate transporter 1 (VGLUT1^+^)] are in close apposition to some *Atoh1*-lineage neurons by immunohistochemistry (Fig. 1*B’–B’’’*; Yuengert et al., 2015; Pop et al., 2022). However, the extent to which *Atoh1*-lineage neurons are mono- or polysynaptically connected to proprioceptive inputs is unknown.

To further our understanding of how CC and *Atoh1*-lineage medial and lateral cells respond to electrical activity and sensory processing, we used modern molecular genetic tools to reproducibly label distinct cell types and a reductionist electrophysiological approach of dissected in vitro spinal cord preparations with whole-cell patch-clamp recordings (Hamill et al., 1981). Overall, we found that CC neurons are large cells and upon current injection show capacity to fire at high frequencies. In addition, CC neurons have a distinctive hyperpolarization-activated current (I_h_) with related sag potential. We also find that *Atoh1*-lineage medial and lateral cells have similar electrophysiological properties, perhaps due to their common developmental lineage, despite their differences in spatial distribution and anatomical projections. In addition, in line with previous reports, we find that 80% of CC neurons receive inputs either mono- or polysynaptically from sensory afferent excitation of the lumbar 2–4 (L2–4) roots (Holmqvist et al., 1956; Osborn and Poppele, 1988). In contrast, very few *Atoh1*-lineage medial and lateral neurons receive lumbar inputs. Instead, *Atoh1*-lineage neurons receive heterogeneous local inputs from sensory afferents and adjacent interneurons in the intermediate spinal cord. Altogether, we find that CC and *Atoh1*-lineage neurons, which are in close proximity in laminae V–VII of the thoracolumbar spinal cord, have unique intrinsic electrophysiological properties and input organization suggesting that they represent discrete circuit modules mediating proprioception.

## Materials and Methods

### Mouse strains

All animal experiments were performed in accordance with the Institutional Animal Care and Use Committee's regulations. The following mouse strains were used: *Gdnf^IRES2-CreERT2^* (JAX #024948; Cebrian et al., 2014), *Atoh1^Cre^* knock-in (Yang et al., 2010), *Atoh1^P2A-FLPo^* (JAX# 038914; Ogujiofor et al., 2021), *Pv^IRES-Cre^* (JAX# 017320; Hippenmeyer et al., 2005), *Hoxa4::Cre* (Huang et al., 2012), *R26^LSL-ChR2(H134R)-EYFP^* (Ai32; Madisen et al., 2012), *R26^doublestop(ds)-HTB^* mouse (Stam et al., 2012; Li et al., 2013), RCE::RFrt (Miyoshi et al., 2010), *R26^FSF^^-^^tdTom^* (Ai65 crossed to germline CRE recombinase to make a FLPo-dependent tdTomato; JAX#021875; Madisen et al., 2015), and *R26^LSL^^-^^tdTom^* (Ai14; JAX #007914; Madisen et al., 2010). *Gdnf^IRES2-CreERT2^* or *Atoh1^Cre^* mice were crossed to *R26^LSL^^-^^tdTom^* mice to label CC and *Atoh1*-lineage neurons with tdTomato (*Gdnf^TOM^* and *Atoh1^TOM^*, respectively). *Atoh1^P2A-FLPo^* crossed to RCE::RFrt, a FLPo-dependent GFP, labels *Atoh1*-lineage neurons with GFP (*Atoh1^GFP^*). *Gdnf^TOM^* pups (aged P7 and/or P8) were injected with 10 mg/ml tamoxifen (Sigma-Aldrich) dissolved in sunflower oil (Sigma-Aldrich) with 10% ethanol at a dosage of 0.1 mg/g pup. All mice were outbred and thus, are mixed strains (at least C57Bl/6J, C57Bl/6N, and ICR).

The *Gdnf^IRES2-CreERT2^* mice label specifically CC neurons in the thoracic to upper lumbar spinal cord and label ∼29% of retrogradely labeled CC neurons (Hantman and Jessell, 2010; Pop et al., 2022). The *Atoh1^Cre^* knock-in mice, which label spinal cord *Atoh1*-lineage cells, become dysregulated in ∼47% of mice (Yuengert et al., 2015). Dysregulated mice are excluded from experiments by detecting tdTomato fluorescence in the skin and mesenchyme of P0–P1 pups. The *Atoh1^Cre^* knock-in mice also ectopically label subsets of motor neurons (Ogujiofor et al., 2021). This is likely due to the transient expression of the CRE recombinase in these neurons and not endogenous *Atoh1* expression because the *Atoh1^P2A-FLPo^* line does not label motor neurons (Ogujiofor et al., 2021). The *Atoh1^P2A-FLPo^* line reliably labels *Atoh1*-lineage neurons without the dysregulation or ectopic motor neuron expression seen in *Atoh1^Cre^* mice. The expression differences in *Atoh1^Cre^* and *Atoh1^P2A-FLPo^* mice may be due to the way they were designed: the CRE recombinase is knocked into the *Atoh1* locus in *Atoh1^Cre^* mice while the FLPo recombinase is inserted in cis at the end of the *Atoh1* locus in *Atoh1^P2A-FLPo^* mice (Yang et al., 2010; Ogujiofor et al., 2021). Consequently, we used the *Atoh1^P2A-FLPo^* mice to avoid any dysregulation in the rabies virus (RV) tracing experiments. Furthermore, to obtain HTB expression using the intersectional *R26^ds-HTB^* strain, we crossed the *Atoh1^P2A-FLPo^* mice to *Hoxa4::Cre*, a strain expressing CRE caudal to the lower medulla and known to be reliably expressed in spinal *Atoh1*-lineage neurons (Huang et al., 2012; Yuengert et al., 2015).

### Electrophysiological recordings

#### Transverse slices

Mice P10–17 were anesthetized with isoflurane (drop technique) and decapitated. A spinal cord section spanning thoracic and lumbar roots T2–L2 was dissected out by performing dorsal and ventral laminectomies using chilled and oxygenated dissecting solution. The dura mater was cut on the midline, and the dorsal and ventral roots were cut close to the spinal cord. The isolated spinal cord section was then embedded in 2.5% low-melt agarose. Afterward, a dissecting solution was then poured into the dissecting chamber and oxygenated with 95% O_2_/5% CO_2_. Spinal cord slices of 300–350 µm thickness were sliced using a Leica Vibratome 1000 Plus. Transverse sections were obtained between T9 and L1. The dissecting solution contained the following (in mM): 81 NaCl, 26 NaHCO_3_, 75 sucrose, 2.5 KCl, 1.25 NaH_2_PO_4_, 0.5 CaCl_2_, 5 MgCl_2_, and 20 dextrose (305–310 mOsm). Afterward, slices were incubated in a slightly modified aCSF solution containing the following (in mM): 130 NaCl, 2.5 KCl, 1.25 NaH_2_PO_4_, 0.75 mM CaCl_2_, 2.5 mM MgCl_2_, 26 NaHCO_3_, and 10 dextrose for 15 min at 34°C and then for at least 45 min at room temperature (RT) until transferred to the recording chamber.

For transverse slice recordings, the aCSF bath solution contained the following (in mM): 130 NaCl, 2.5 KCl, 1.25 NaH_2_PO_4_, 2 CaCl_2_, 1.25 MgCl_2_, 26 NaHCO_3_, and 10 dextrose. Whole-cell patch clamp was performed using borosilicate capillaries pulled with a P-87 flaming-brown micropipette puller (Sutter Instrument Company). The pipettes had a resistance of 3–6 MΩ, when using an internal solution containing the following (in mM): 134 KCl, 10 NaCl, 1 CaCl_2_, 1 MgCl_2_, 1 dextrose, 4 Na_2_ATP, and 10 HEPES-K (pH 7.25, 290–295 mOsm). A junction potential of 11 mV was determined empirically and was consistent with that calculated theoretically by using the corresponding function in Clampfit. This value was subtracted to adjust the voltages reported herein. Recordings were obtained using a 700B multiclamp amplifier (Molecular Devices). Neurons were visualized using a Nikon EF600N Eclipse microscope equipped with infrared differential interference contrast, a CCD camera, and epifluorescence. A fluorescence filter set Y-2E/C (Texas Red, EX 540–580, DM 595, BA 600–660) was used to visualize tdTomato labeled neurons. Acquisition was done at 10 kHz, and data were analyzed offline using Clampfit Analysis Suite (Molecular Devices), GraphPad Prism 9, and Microsoft Excel 2015.

After achieving whole-cell configuration, cells were held near resting potential (V_m_rest) for at least 5 min to allow for dialysis of the pipette internal solution. Afterward, when needed, cells were held at −70 mV by injecting current of the appropriate sign and amplitude. Depending on the experiment, one or several of the following protocols were applied: For rheobase evaluation, first, a gross determination of action potential (AP) firing was made using 10–20 pA incremental steps (1 s duration), and then a baseline subthreshold current with incremental steps of 1–3 pA in successive sweeps was used. The rheobase was considered the step where the first action potential (AP) was fired and where APs were consistently fired in the following steps. To determine firing properties and firing types, we applied 10 Δ50 pA/1 s current steps (range, 50–500 pA). Neurons were classified as pertaining to one out of four firing-type groups: tonic (T), fading (F), single (S), or undefined (U). U cells were classified as such because their firing pattern changed depending on the intensity of the stimulus. T-firing–type cells showed increasing firing frequency and stable AP amplitude all along the ten steps. Cells were classified as F-firing type if the amplitude of three of the last APs fired in a step was reduced by 25% or more compared with the first AP in that step and subsequent sweeps showed an even more pronounced reduction in the average AP amplitude. The F-firing type also presented with AP failure during a sweep, from which they could not recover (i.e., they did not resume firing APs) and a rise in the baseline V_m_ in subsequent APs. To determine the presence of hyperpolarization-activated currents (I_h_) in voltage clamp, and of sag potentials and rebound AP firing in current clamp, cells were held at −70 mV and hyperpolarizing potentials (nine Δ-10 mV steps/1 s), or hyperpolarization currents (up to 20 steps of Δ-50 pA/200 ms) were applied. To confirm the presence of hyperpolarization-activated cyclic nucleotide–gated (HCN) channels, we used 50 µM of ZD7288 to inhibit both I_h_ and sag potentials.

#### Spinal cord sagittal hemisections

A spinal cord section from T2 to L6 was dissected out as described for transverse slices. Dorsal roots L2–L4 and T11–T13 roots were preserved for afferent stimulation. The dura mater was cut on the midline with scissors, and the arachnoid and the pia mater were cut using a 21-gauge needle. The left and right spinal cord sides were then separated to obtain sagittal hemisections. After incubation in the modified aCSF solution for 15–30 min at 34°C, hemisections were placed in a submersion-recording chamber with the midline facing up. Recordings were then performed in oxygenated aCSF superfused at a rate of 2–3 ml/min, and, in most of the experiments, 100 µM picrotoxin (Alomone Labs) and 0.5–1 µM strychnine (Sigma-Aldrich) were included. The internal solution was the same as above with 2–4 mM QX314 to block Na^+^ channels from driving AP firing. In this recording configuration, only CC neurons in the *Gdnf^TOM^* mouse model or medial neurons in the *Atoh1^TOM^* mouse model were at depths suitable for recordings. Lateral cells are too deep to be recorded using this approach.

#### Spinal cord horizontal sections

Given that the optical depth limit in sagittal hemisection preparations precludes recording from *Atoh1*-lineage lateral cells, a spinal cord horizontal preparation was used to record from this cell group. A spinal cord section from T2 to L6 was dissected out as described for other preparations. One dorsal root between L2 and L4 and one dorsal root between T11 and T13 were preserved for afferent stimulation. To target only the area of interest without damaging the dorsal roots, the spinal cord was rested on a short block of high-melt agarose with the ventral part facing up and removed using a vibratome. The agarose block spanned a segment from T8 to L2, so segments outside of these areas were not reached by the blade. In this configuration, CC and *Atoh1*-lineage medial and lateral cell types are accessible for recordings. Even though we focused on the *Atoh1*-lineage lateral population for this preparation, recordings were also done on CC and *Atoh1*-lineage medial neurons. No overt difference seems to exist in the afferent responses onto these cells between the sagittal hemisection and horizontal spinal cord preparations under the recording conditions performed for this work.

#### Afferent stimulation

Recordings were obtained between segments T10–L2 by performing a whole-cell patch clamp. Suction electrodes (A-M Systems) were used to stimulate the lumbar dorsal roots (L2–L4) and lower thoracic dorsal roots (T11–13). Fluorescently (tdTomato or eGFP) labeled cells were recorded in a whole-cell configuration between segments T10 and L2 while stimulating dorsal roots near the recording site (T11–13, local) or several segments caudal (L2–L4, distal). For a rough estimation of the distance traveled from the dorsal root entry site to the recorded cell, the separation (in mm) between a cut just proximal of the L2 and L4 dorsal root ganglia (DRG) and the dorsal root insertion at T11 was determined in three individuals. L2 to T11 separation was 6.8 ± 0.29 mm, and L4 to T11 was 9.8 ± 0.29 mm, *n* = 3. The threshold was determined by gradually increasing the stimulation intensity until at least three out of five stimulation attempts yielded a synaptic response. Using an A365 stimulus isolator (WPI), 0.2 ms electric shocks of increasing magnitude were applied starting at ∼5 µA and up to 5 mA. To determine the evoked EPSC (eEPSC) response type, we exposed most cells to stimulation intensities of 25, 100, and 500 µA or at suprathreshold stimulations of 1.5× to 2× threshold. In addition, 5 mA intensity was used as a top limit and was reached only in recordings where cells did not respond to lower stimulation intensities. In recordings where cells showed a response, the highest thresholds reached during thoracic dorsal root stimulation were 1.9 mA for CC cells, 1.7 mA for medial cells, and 1.2 mA for lateral cells and during lumbar dorsal root stimulation were 2.3 mA for CC cells, 0.3 mA for medial cells, and 3.4 mA for lateral cells. Stimulation at 5 mA did not appear to cause nonspecific excitation of neurons in our preparation for three reasons: (1) patching neighboring cells in the same preparation as nonresponsive (NR) cells elicited an EPSC indicating that the nonresponsive cell was not aberrantly activated by the high stimulation. (2) Cells that were nonresponsive to thoracic dorsal root stimulation would sometimes be responsive to lumbar dorsal root stimulation, which was farther away, indicating that the recorded cells were not aberrantly activated by high stimulation at a dorsal root closer to the cell. (3) Some neurons that had a single EPSC at a low threshold did not change their EPSC shape or amplitude even up to stimulation intensities in the milliamp range, indicating that increased stimulation intensity did not cause aberrant excitation.

#### Optogenetic stimulation

Channelrhodopsin (ChR2) was expressed in parvalbumin (*Pv*)-lineage sensory neurons [*Pv^IRES-Cre^*; *R26^LSL-ChR2(H134R)-EYFP^* (Ai32)] and tdTomato in *Atoh1*-lineage neurons (*Atoh1^P2A-FLPo^*; *R26^FSF-tdTom^*). Transverse slices were prepared as described above from P10 to P14 mice to avoid expression of ChR2 in PV^+^ spinal cord neurons. For optogenetic stimulation, blue light (465–495 nm) from an X-Cite 200 DC (Lumen Dynamics) lamp was used. The voltage pulse to activate the shutter had a time threshold of 1.6–2.4 ms. The shutter itself has an average 6 ms latency to open and an average 6 ms latency to close. Therefore, light stimulation-to-oEPSC (optogenetic EPSC) latencies should be considered as apparent latencies. The latency to oEPSC was determined for each cell from the onset of the electrical pulse to open the shutter (7 sweeps/cell for *Atoh1*-lineage medial cells, 10 sweeps for *Atoh1*-lineage lateral cells). The standard deviation of the latencies is the jitter value for each cell.

### Experimental design and statistical tests

Raw traces were analyzed using Clampfit (Molecular Devices). All data and graphs were processed in Microsoft Excel 2015 and GraphPad Prism 9. Mean ± SEM are reported throughout the manuscript. *p* values are included in the figures. Statistical tests used are detailed in the Results section and/or figure legends. Welch's *t* tests were used because almost all parameters tested showed non-normal distributions or were compared between groups with unequal variances.

### Transsynaptic rabies virus tracing

Rabies virus (RV) ΔG mCherry-EnvA [Salk Institute Viral Core; 150–200 nl of 8.77 × 10^7^ to 1.38 × 10^9^ TU (transducing units)/ml] was injected in the lower thoracic to upper lumbar (T8–L3) spinal cord of *Hoxa4::Cre; Atoh1^P2A-FLPo^; R26^ds-HTB^* mice at age P8–P10 and harvested 5 d later. Percent infection efficiency (GFP^+^mCherry^+^/total GFP^+^) and percent of presynaptic neurons in the spinal cord (mCherry^+^-only/total mCherry^+^) were counted in spinal cord sections that were infected with RV ΔG mCherry-EnvA (9–29 sections/mouse, *n* = 5 mice).

### Histology

Mice were anesthetized with Avertin (2,2,2-tribromoethanol; 0.025–0.030 ml of 0.04 M Avertin in 2-methyl-2-butanol and distilled water/g mouse) and transcardially perfused with 0.012% w/v heparin/PBS and then 4% paraformaldehyde (PFA)/PBS. A dorsal laminectomy exposed the spinal cord, and the tissue was fixed for 2 h. The tissue was washed with PBS and cryoprotected in 30% sucrose/H_2_O. Spinal cords were embedded in OCT (Tissue-Tek Optimal Cutting Temperature compound) and sectioned on a Leica Microsystems CM1950 Cryostat. Cryosections (20–30 µm) were blocked with 1% normal goat serum (Jackson ImmunoResearch Laboratories)/0.3% Triton X-100 (Sigma-Aldrich) in PBS for 1 h at room temperature (RT) and incubated overnight with primary antibodies at 4°C. Sections were washed in PBS and incubated for 1 h at RT with secondary antibodies. Sections were washed in PBS and mounted with Aqua-Poly/Mount (Polysciences). The primary antibodies included 1:500 chicken anti-GFP (Aves Labs), 1:500 rabbit anti-dsRed (Clontech), 1:500 mouse anti-NEUN (MilliporeSigma), 1:1000 rabbit anti-PVALB (Swant, P27), 1:5,000 guinea pig anti-VGLUT1 (MilliporeSigma), 1:500 rabbit anti-NF200 (Sigma-Aldrich), and 1:500 goat anti-TRKC (R&D Systems). The secondary antibodies included 1:500 goat anti-chicken Alexa 488; 1:500 goat anti-rabbit Alexa 488, 568, or 647; and 1:200 goat anti-guinea pig Alexa 647 (Invitrogen). Fluorescent images were taken on a Carl Zeiss LSM880 confocal microscope with the 20× air objective. Images were pseudocolored using Adobe Photoshop 2023 (Adobe) or Fiji (Schindelin et al., 2012).

### RNAscope

RNAscope Fluorescent Multiplex Assay version 1 (v1) or 2 (v2; Advanced Cell Diagnostics) was performed following the manufacturer's instructions. Paraformaldehyde-fixed *Gdnf^Tom^* P30 spinal cord sections (20 µm) were processed using the following probes: *Hcn1* (ACDBio, #423651), *Hcn2* (ACDBio, #427001-C3), *Hcn3* (ACDBio, #551521-C3), and *Hcn4* (ACDBio, #421271-C3). Incubation steps were performed in the HybEZ II oven set to 40°C. The only deviations from the manufacturer's protocols were to use protease III for 3 min for v1 and 10 min for v2 with no antigen retrieval step. RNAscope was followed by immunohistochemistry with 1:500 rabbit anti-dsRed (Clontech; primary antibody) and 1:500 goat anti-rabbit Alexa 568 (Invitrogen; secondary antibody). Slides were mounted in Aqua-Poly/Mount (Polysciences) and coverslipped.

## Results

### Distinct passive and active properties of Clarke's column (CC) neurons and *Atoh1*-lineage medial and lateral neurons

CC (*Gdnf^TOM^*) and *Atoh1*-lineage (*Atoh1^TOM^* or *Atoh1^GFP^*) cells are excitatory neurons present in laminae V–VII of the spinal cord in nonoverlapping populations (Fig. 1*A–C*; Hantman and Jessell, 2010; Yuengert et al., 2015). CC and some *Atoh1*-lineage neurons (medial and lateral) have proprioceptive afferent terminals (PV^+^ VGLUT1^+^) in close apposition to their soma (Fig. 1*A’,A’’,B’–B’’’*). We focused on a comprehensive electrophysiological characterization of CC, *Atoh1*-lineage medial, and *Atoh1*-lineage lateral cell groups. We did not record cells along the boundary between the *Atoh1*-lineage medial and lateral groups to avoid misassignment. The basic excitability properties of CC, *Atoh1*-lineage medial, and *Atoh1*-lineage lateral cells were determined utilizing the whole-cell patch-clamp approach in transverse slices that are 300–350 µm thick. To test their baseline activity, no holding current was applied so that the cell was recorded at its resting membrane potential, V_m_rest. Examples of spontaneously firing cells at V_m_rest and of the rheobase determination are shown (Fig. 1*D*, top and bottom panels, respectively). CC neurons show a four- to eightfold larger capacitance than *Atoh1*-lineage medial and lateral cells (CC, 138.1 ± 0.11 pF, *n* = 36; medial, 19.35 ± 1.95 pF, *n* = 23; and lateral 30.9 ± 2.90 pF, *n* = 30. Welch's *t* tests: CC vs medial, *p* < 0.0001; CC vs lateral, *p* < 0.0001; and medial vs lateral, *p* *=* 0.0025). We also find differences in input resistance (Rm; CC, 0.41 ± 0.10 GΩ, *n* = 38; medial, 1.81 ± 0.31 GΩ, *n* = 24; and lateral 0.99 ± 0.212 GΩ, *n* = 29. Welch's tests: CC vs medial, *p* = 0.0004; CC vs lateral, *p* = 0.0163; and medial vs lateral, *p* *=* 0.0426) and resting potential (V_m_rest; CC, −58.91 ± 1.43 mV, *n* = 35; medial, −53.75 ± 0.98 mV, *n* = 24; and lateral −51.9 ± 1.72 mV, *n* = 29. Welch's tests: CC vs medial, *p* = 0.0045; CC vs lateral, *p* = 0.0028; and medial vs lateral, ns; Fig. 1*E*). Roughly 45% of CC cells spontaneously fire action potentials (APs) at rest while 65% and 40% of *Atoh1*-lineage medial and lateral cells fire spontaneously. No differences in spontaneous firing frequency were found (Fig. 1*E*). To test for excitability, we determined the rheobase. Consistent with the difference in capacitance and internal resistance, CC and *Atoh1*-lineage lateral neurons show larger rheobase than *Atoh1*-lineage medial cells (CC, 81.83 ± 10.4 pA, *n* = 35; medial, 32.6 ± 4.79 pA, *n* = 24; and lateral 68.15 ± 11.15 pA, *n* = 27. Welch's tests: CC vs medial, *p* = 0.0001; CC vs lateral, ns; and medial vs lateral, *p* = 0.0075; Fig. 1*E*). Cells with larger capacitance tend to have low input resistance. We found that only lateral cells showed a negative correlation (Pearson’s correlation, *r*) between input resistance and cell capacitance (CC, 0.02; medial, 0.17; and lateral, −0.45). In addition, we found a negative correlation between rheobase and input resistance (CC, −0.3123; medial, −0.48; lateral, −0.38) consistent with cells having high input resistance being more excitable at lower stimulation intensities.

**Figure 2.**
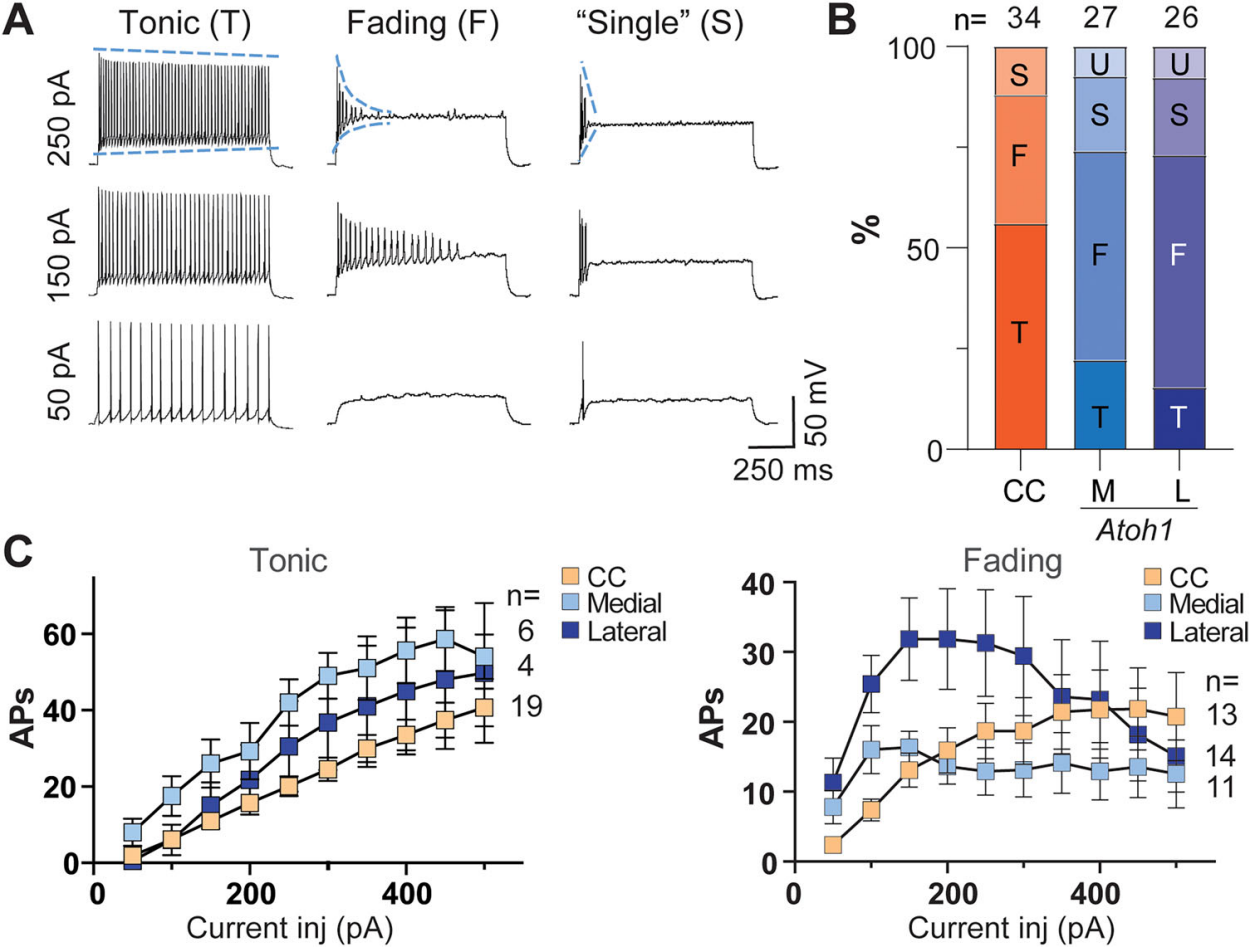
Firing types for CC and *Atoh1*-lineage medial and lateral neurons. ***A***, Current steps in increments of 50 pA were injected up to 500 pA. Steps at 50, 150, and 250 pA are shown. The columns show the main firing types found for all cell groups. The tonic-firing type (T) showed an increase in frequency at higher steps. The fading-firing type (F) showed increased firing frequency upon the first few depolarization steps, but further depolarization failed to sustain firing. The “single”-firing type (*S*) showed less than three APs, even at the highest currents injected. The blue lines on the top traces highlight the slope differences among the firing types, namely, the decrease in maximal AP amplitude over the time course of the current injection and the degree of baseline depolarization. The slope is shallow for the T-firing type but very steep for F- and *S*-firing cells. ***B***, Percentages of firing types for each cell group. M and L neurons that changed firing type depending on the level of current injection were classified as undefined (U). ***C***, The relation for current injected and # of APs fired is shown for T- and F-firing types for each cell group.

Next, we determined the firing properties of these neurons subjected to discrete increases in intracellular current injections. Upon 10 pulse steps at 50 pA increments, the firing properties were classified in either of four groups: tonic (T), fading (F), single (S), or undefined (U; see Materials and Methods for classification criteria). The T-firing type was stable across the stimulation spectrum with little to no accommodation. CC T-firing–type cells respond linearly to current injection (Fig. 2*C*). The few *Atoh1*-lineage neurons that are of the T-firing type are reported for reference (Fig. 2*C*). In contrast, the baseline became clearly depolarized in the F-firing type with larger amounts of current injected (Fig. 2*A*), resulting in eventual firing failure during the 1 s step. This phenomenon may be due in part to depolarization block where suboptimal repolarization results in a slower recovery from inactivation of fast voltage-dependent Na^+^ channels (Grace and Bunney, 1986; Richards et al., 1997; Qian et al., 2014). Regardless of the underlying cause, the F-firing type is present in a larger proportion of *Atoh1*-lineage neurons in our recording conditions. CC and *Atoh1*-lineage medial and lateral F-firing–type cells had different kinetics of AP firing upon current injection (Fig. 2*C*, fading). CC F-firing–type cells reached a plateau ∼450 pA, while *Atoh1*-lineage medial F-firing–type cells reached a plateau ∼100 pA and lateral F-firing–type cells peaked at ∼200 pA and then decreased with increasing current injection (Fig. 2*C*). The *S*-firing type was defined as cells that had <3 APs at all current injections. The *S*-firing type seems an extreme case of F-type firing, where the decrease in AP amplitude is very steep and firing fails quickly (Fig. 2*A*, compare dashed blue lines among cell types).

The proportion of firing types differs among groups (Fig. 2*B*). T-firing type is predominant in CC cells, whereas it is less prevalent in medial and lateral *Atoh1*-lineage cells (CC, 56%; medial, 22.2%; and lateral, 15.4%). In contrast, the F-firing type is more common in *Atoh1*-lineage cells (CC, 32%; medial, 51.9%; and lateral, 57.7%). The *S*-firing type is present in lower numbers in all the groups (CC, 12%; medial, 18.5%; and lateral, 19.2%). Interestingly, some cells in medial and lateral groups switched among different firing types depending on the strength of the stimulation and were categorized as U cells. Similarly, it has previously been shown that some neurons can switch from tonic firing to burst firing under the influence of neuromodulators (Grace and Bunney, 1984a,b; Zhang, 2003), suggesting that firing-type classification is not absolute and may better be interpreted as the predominant physiological state of a neuron under those experimental conditions and developmental stage.

### Hyperpolarization-activated I_h_ currents and sag potentials are present in CC cells

**Figure 3.**
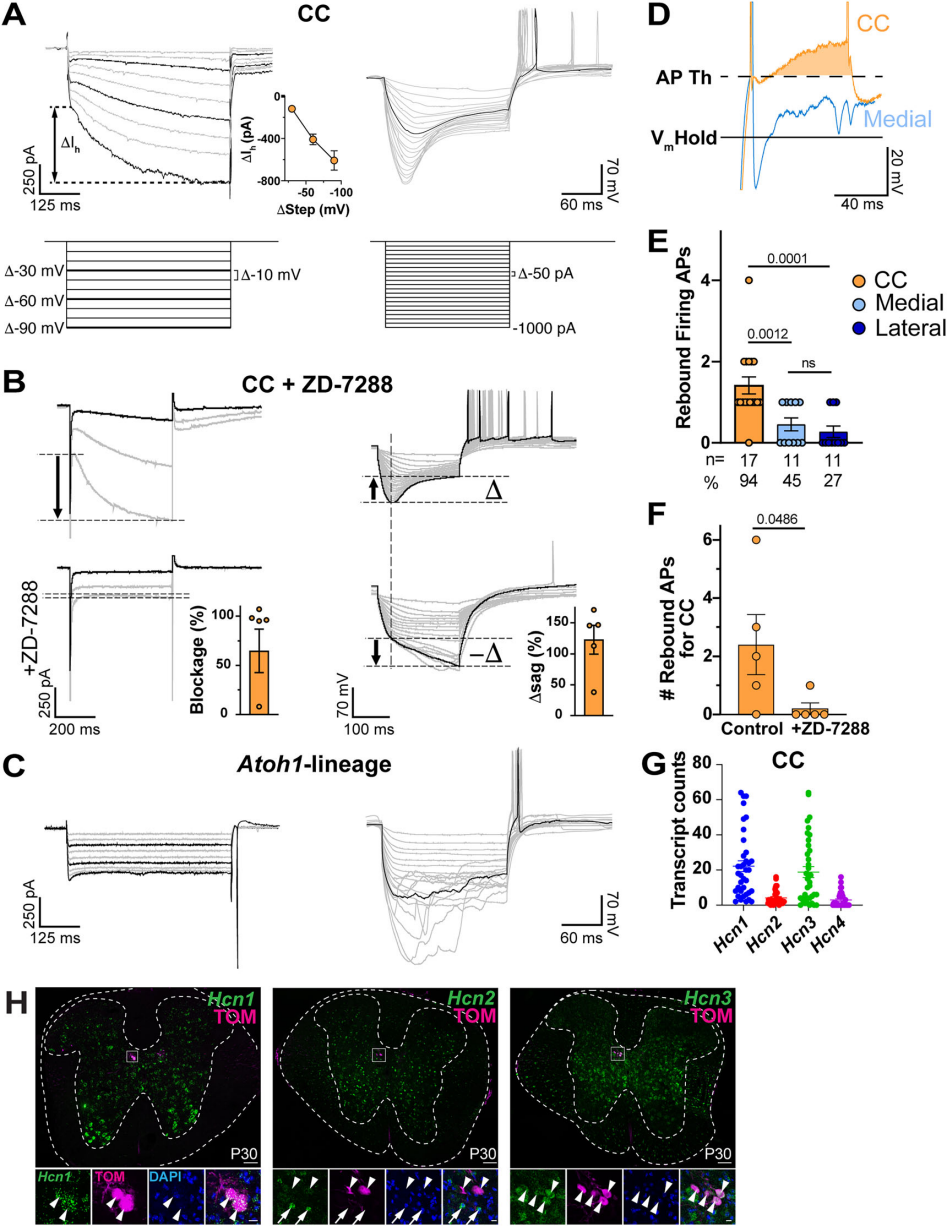
I_h_ currents and sag potentials are present in CC cells. ***A***, Hyperpolarizing −10 mV voltage steps (left) and −50 pA current steps (right) induced I_h_ currents and sag potentials in CC cells. The inset in the middle shows the quantification for the amplitude of the I_h_ current induced by steps at −30, −60, and −90 mV, after the subtraction of the instantaneous leak current. ***B***, To confirm the nature of the hyperpolarization-activated currents in CC neurons, we used ZD-7288 to block I_h_ currents. Except for one cell, a nearly complete blockage of the hyperpolarization-activated current was achieved with an average 70% blockage (left traces, bar graph to the right). The sag potential (Δ) is blocked with ZD-7288 and membrane hyperpolarization continues (−Δ; right traces). The difference in sag potential is >100% compared with control when I_h_ is blocked (right traces, bar graph to the right). ***C***, Neither I_h_ currents nor sag potentials were induced in *Atoh1*-lineage neurons. Instead, *Atoh1*-lineage neurons showed high susceptibility to damage when subjected to hyperpolarization (right). ***D***, Compared to *Atoh1*-lineage medial neurons, CC cells showed a relatively large after depolarization (orange-shaded area) following the first rebound AP fired. ***E***, Number of rebound APs fired following a hyperpolarization step. ***F***, Blockage of I_h_ current in CC cells almost eliminates rebound firing, strongly suggesting that upon repolarization, residual I_h_ depolarizes CC cells inducing rebound firing. Paired one-tailed *t* test: 0.0486. ***G***, Transcript counts for *Hcn1*, *Hcn2*, *Hcn3*, and *Hcn4* transcripts in CC neurons from single-cell RNA-seq data published in Baek et al. (2019). ***H***, *Hcn1* and *Hcn3* mRNA transcripts are present in CC neurons (RNAscope^+^TOM^+^, arrowheads, left and right panels). *Hcn2* mRNA is weakly expressed in CC neurons (RNAscope^+^TOM^+^, arrowheads, middle panel), while nearby neurons express *Hcn2* mRNA (arrows). Scale bars: 100 µm (***H***), 10 µm (***H***, insets).

When subjected to hyperpolarizing voltage or current steps, CC cells present the activation of a current that resembles I_h_ in voltage clamp and sag potentials in current clamp (Fig. 3*A*). We injected hyperpolarizing current from a baseline of −70 mV (Fig. 3*A*). The rate of activation and amplitude of I_h_ currents increased with each voltage step. I_h_ current increased with increasing hyperpolarization (Fig. 3*A*, inset on the right side, −118 ± 20.6 pA at Δ-30 mV, −408 ± 50.3 pA at Δ-60 mV, and −616 ± 81.5 pA at Δ-90 mV, *n* = 10). Similarly, current steps of successive −50 pA increments induced sag potentials that increased in rate and amplitude with increased hyperpolarization (Fig. 3*A*, right side).

To confirm that the currents and sag potentials activated during hyperpolarization stimuli were due to I_h_, CC cells were exposed to an I_h_ blocker, ZD-7288 (50 µM; Gasparini and DiFrancesco, 1997). Four out of five cells showed strong I_h_ blockage of near 100% (overall blockage, ∼70%; Fig. 3*B*, left panels). For sag potentials, the hyperpolarization of the plasma membrane continued its hyperpolarizing course upon I_h_ inhibition, instead of reverting direction as for sag potentials (represented by the arrow in the opposite direction as in the control and the −Δ sign in the traces in the bottom right). Therefore, quantification of the sag Δ [defined as (Δ− −Δ)/Δ] yielded a delta difference greater than 100% in cells with strong I_h_ blockage (Fig. 3*B*, right panels).

In contrast, *Atoh1*-lineage neurons did not evidence an I_h_ current upon hyperpolarizing voltage or current steps (Fig. 3*C*). *Atoh1*-lineage neurons were more susceptible to damage due to hyperpolarization. If injected with a current more negative than −120 mV, these cells showed anomalous potential transitions that resemble irregular step depolarizations (Fig. 3*C*, right side). Often, the usually high Rm was lost, suggesting plasma membrane leakage and damage.

After the hyperpolarization step, some cells had rebound firing upon repolarization. A closeup of the rebound firing in a CC cell reveals that the AP is mounted on a transient depolarization that may facilitate repetitive firing after rebound that is not present in *Atoh1*-lineage neurons (Fig. 3*D*, orange-shaded area). The maximum number of APs fired after rebound was higher in CC neurons (CC, 1.44 ± 0.21 APs, *n* = 17; medial, 0.455 ± 0.16 pA, *n* = 11; and lateral, 0.27 ± 0.14 pA, *n* = 11. Welch's tests: CC vs medial, *p* = 0.0012; CC vs lateral, *p* = 0.0001; and medial vs lateral, ns; Fig. 3*E*). Rebound firing was more common in CC cells with 94% of CC cells (16/17), 45% of medial cells (5/11), and 27% of lateral cells (3/11) showing rebound firing with a hyperpolarization threshold to rebound firing approximately −140 mV for all cell types (Fig. 3*E*). *Atoh1*-lineage neurons never had rebound firing more than one AP. Interestingly, rebound AP firing in CC cells was lost upon application of the I_h_ inhibitor ZD-7288, strongly suggesting that AP rebound firing in these cells depends on I_h_ (Fig. 3*F*).

I_h_ is driven by a family of hyperpolarization-activated cyclic nucleotide–gated (HCN) ion channels (Pape and McCormick, 1989; Ludwig et al., 1998). To examine which HCN channels are expressed in CC neurons, we analyzed single-cell RNA sequencing data of spinocerebellar neurons in aged P6–P7 mice from Baek et al. (2019). Cluster SCT5 in their dataset expresses markers consistent with CC neurons. An analysis of transcript counts in 37 cells of cluster SCT5 found HCN1 and 3 to be enriched (Fig. 3*G*). We then performed an RNAscope of *Hcn1*, *Hcn2*, *Hcn3*, and *Hcn4* in spinal cords from *Gdnf^TOM^* mice that label CC neurons. We found *Hcn1* and *Hcn3* mRNA transcripts enriched in CC neurons with *Hcn2* weakly expressed (Fig. 3*H*), consistent with the single-cell transcriptomic data. We did not detect any *Hcn4* mRNA in the spinal cord, which we attribute to technical difficulties as *Hcn4* has previously been detected in rodent spinal cord (Tu et al., 2004; Hughes et al., 2012; Nakagawa et al., 2020).

### Afferent inputs to the intermediate spinal cord

**Figure 4.**
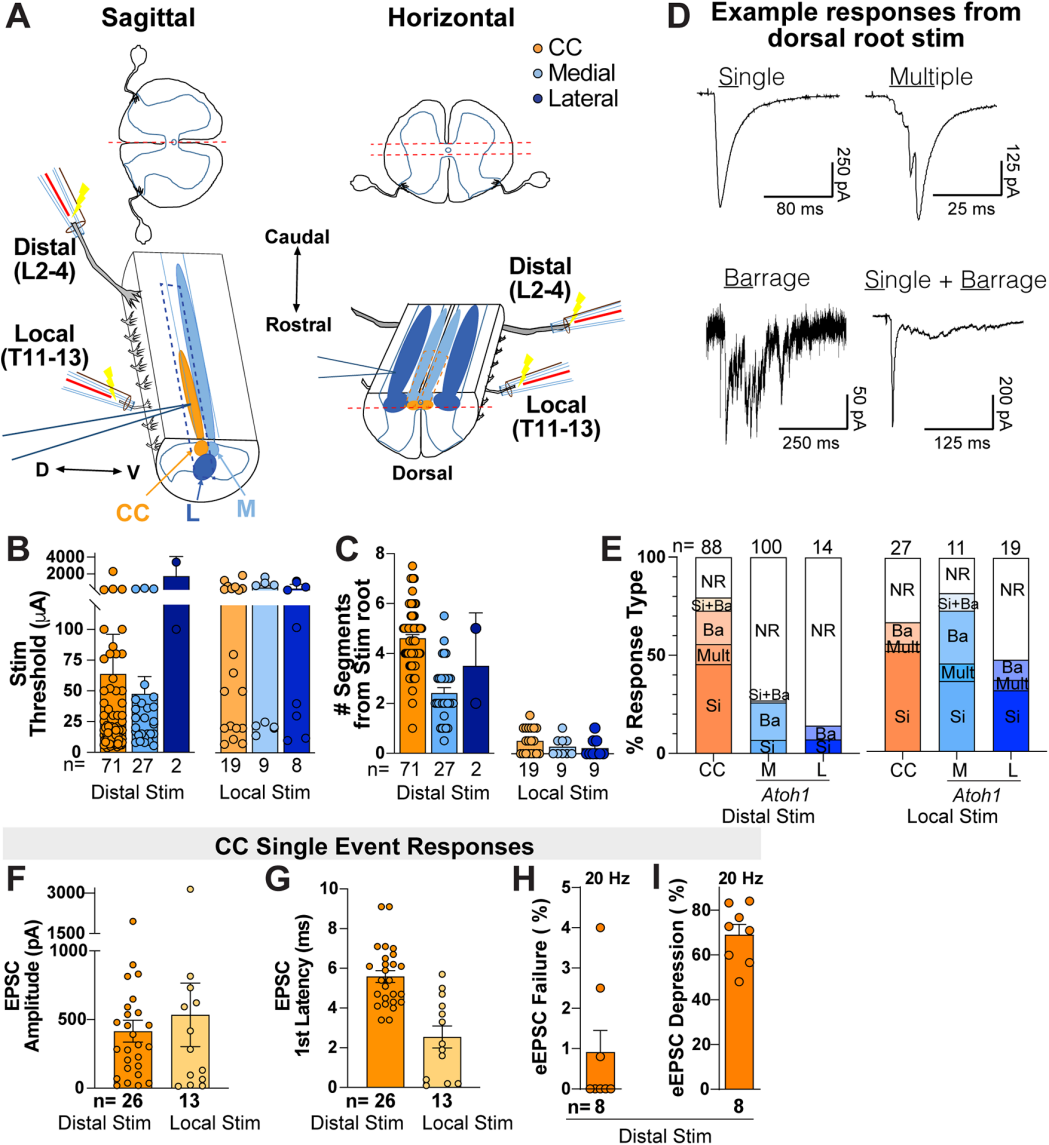
Organization of primary afferent inputs to CC and *Atoh1*-lineage medial neurons in the intermediate spinal cord. ***A***, Schematic of electrophysiological recordings in sagittal hemisected (left) or horizontally sectioned (right) spinal cords. Stimulation suction electrodes were used either at the lumbar L2–L4 roots or the thoracic T11–T13 roots while recording from CC or *Atoh1*-lineage neurons in spinal cord segments T10–L2. ***B***, ***C***, The overall threshold stimulation to any response type and the number of segments between the recorded cell and stimulated dorsal root are shown for both lumbar (distal) or thoracic (local) dorsal root stimulation. These include cells that responded to any stimulation intensity. In general, thresholds were higher upon local stimulation for all cell types. ***D***, Samples of the four types of evoked EPSCs recorded upon dorsal root stimulation: Single, multiple, barrage, and Si + Ba. Note that the multiple category has eEPSCs whose peak amplitudes are within 10 ms of stimulation and the barrage category has several eEPSCs that occur within hundreds of ms from stimulation. The representative Ba sample was taken from an M cell. All other examples are from CC cells. ***E***, Percentages for each type of eEPSC for CC and *Atoh1*-lineage M and L cells. Nonresponsive (NR) cells are included. Upon lumbar stimulation (distal), Si eEPSCs were the most frequent type in CC cells, followed by Ba eEPSCs. Only 27% of M cells and 14% of the L cells showed any response (i.e., 73 and 86% were NR, respectively). No Multi eEPSCs were present in M cells. Only two L cells responded and were either the Si or Ba type. Upon thoracic stimulation (local), CC and *Atoh1*-lineage medial neurons had several responsive cells (CC, 67%; medial, 82%), whereas 47% of *Atoh1*-lineage lateral neurons were responsive to local stimulation. ***F–I***, Characteristics of CC single eEPSCs. ***F–G***, Amplitude and first latency for Si eEPSCs are shown for distal (lumbar) or local (thoracic) stimulation. ***H***, Si eEPSCs in CC cells did not show any significant failures when stimulated at a frequency of 20 Hz. ***I***, When amplitudes of the first versus the last EPSC in a train were compared, most of them showed depression above 50%.

CC neurons are known to integrate and relay whole hindlimb proprioceptive information (Oscarsson, 1965; Bosco and Poppele, 2001; Hantman and Jessell, 2010), but the inputs to *Atoh1*-lineage neurons are less clear. To test whether both CC and *Atoh1*-lineage neurons receive inputs from the lower thoracic or lumbar spinal cord, we recorded electrically driven afferent signals in vitro in sagittal hemisected or horizontal spinal cord preparations (Fig. 4*A*). In the sagittal preparation, only CC and *Atoh1*-lineage medial neurons were recorded because the *Atoh1*-lineage lateral cells were too deep to be accessible from the midline. In the horizontal preparation, CC and *Atoh1*-lineage medial and lateral neurons were recorded. In both preparations, one local (T11–T13) and/or one distal (L2–L4) dorsal roots were stimulated while recording from CC, *Atoh1-*lineage medial, or *Atoh1*-lineage lateral neurons from the thoracolumbar region (T10–L2).

We found that CC neurons received afferent input from both distal (L2–L4) and local (T11–T13) dorsal roots, while *Atoh1*-lineage medial and lateral neurons received afferent input from mostly local (T11–T13) dorsal roots. Stimulation thresholds from 5 µA to 5 mA were determined for all cells (Fig. 4*B*). Note that stimulation thresholds of 5 mA were reached only when cells were nonresponsive (NR). For L2–L4 (distal) root stimulation, the thresholds were on average: CC, 63.8 ± 32.28 µA, *n* = 71; medial, 40.2 ± 12.20 µA, *n* = 27; and lateral, 1,750.0 ± 1,650.00 µA, *n* = 2. For T11–13 root (local) stimulation, the thresholds were as follows: CC, 303.3 ± 115.18 µA, *n* = 19; medial, 528.9 ± 219.84 µA, *n* = 8; and lateral, 354.9 ± 161.33 µA, *n* = 8 (Fig. 4*B*). We found that stimulating the L2–L4 dorsal roots resulted in afferent responses in CC cells up to seven segments rostral to the excited dorsal root (Fig. 4*C*). On average, we found responsive CC neurons up to 5–6 segments away from the distal (L2–L4) stimulated roots (responsive cells, 4.59 ± 0.15 segments, *n* = 71; nonresponsive cells, 5.97 ± 0.27 segments, *n* = 17, *p* < 0.0001) as well as local (T11–T13) stimulated roots (0.47 ± 0.12 segments, *n* = 16). In contrast, *Atoh1*-lineage neurons were responsive to more local dorsal root stimulation (Fig. 4*C*; for L2–L4 stimulation, medial, 2.56 ± 0.23 segments, *n* = 27, and lateral, 3.50 ± 1.50 segments, *n* = 2, and for T11–13 stimulation, medial, 0.31 ± 0.13 segments, *n* = 9, and lateral, 0.32 ± 0.10 segments, *n* = 9).

We found four different response types (Fig. 4*D*): (1) single (Si), a single evoked excitatory postsynaptic current (eEPSC) upon stimulation of a dorsal root; (2) multiple (Multi), 2–4 discrete eEPSCs whose peak amplitudes are within 10 ms of stimulation; (3) barrage (Ba), several eEPSCs that occur within hundreds of ms from stimulation, possibly due to polysynaptic inputs, burst firing of an input, or asynchronous release from presynaptic terminals; and (4) Si + Ba, a combination of a single eEPSC (usually of larger amplitude) and a barrage of inputs (of lower amplitude). Upon distal root stimulation, 46% of CC neurons received Si inputs, 10% Multi, 17% Ba, and 7% Si + Ba, and 20% were NR. For *Atoh1*-lineage medial neurons, 7% received Si inputs, 19% Ba, and 1% Si + Ba, and 73% were NR. For lateral neurons, 7% received Si inputs and 7% Ba, and 86% were NR (Fig. 4*E*). Upon local stimulation, CC neurons received 52% Si inputs, 4% Multi, and 11% Ba and 33% were NR. For *Atoh1*-lineage medial neurons, 37% received Si inputs, 9% Multi, 27% Ba, and 9% Si + Ba, and 18% were NR. For lateral neurons, 32% received Si inputs, 5% Multi, and 10% Ba, and 53% were NR (Fig. 4*E*). In total, for distal stimulation, 80% of CC neurons (71/88 cells), 27% of medial cells (27/100 cells), and 14% (2/14 cells) showed a response to any stimulation intensity (Fig. 4*E*). For local stimulation, 67% (18/27 cells) of CC neurons, 82% (9/11 cells) of medial cells, and 47% (9/19 cells) for lateral cells showed response to any stimulation intensity (Fig. 4*E*).

*Atoh1*-lineage medial neurons had a high proportion (73%) of cells that were nonresponsive (NR) to lumbar afferent stimulation even as high as 5 mA. To test whether the lack of response was due to the absence of connectivity or due to dorsal root damage, we examined the afferent response of neighboring non–*Atoh1*-lineage neurons in the same preparation. Most of the non–*Atoh1*-lineage neurons (17/19) showed response upon lumbar afferent stimulation and had thresholds of 26.0 ± 7.72 pA, *n* = 17 (data not shown). This indicates that dorsal roots were intact and not damaged and that the lack of response was due to an absence of connectivity between the distal root and *Atoh1*-lineage medial neurons.

We report here the characteristics of the Si eEPSC events in CC neurons (EPSC amplitude: distal stim, 415.3 ± 79.8 pA, *n* = 26; local stim, 534.1 ± 231.5 pA, *n* = 13. EPSC first latency: distal stim, 5.6 ± 0.32 ms, *n* = 26; local stim, CC, 2.5 ± 0.55 ms, *n* = 13; Fig. 4*F,G*). Two pieces of evidence suggest that the distal root stimulation Si eEPSC inputs to CC neurons are monosynaptic. First, a subset of CC Si inputs was subjected to high-frequency stimulation. These showed a small latency variance with a low incidence of eEPSC (evoked EPSC) failures (only 0.9 ± 0.19% failures from a 1 s, 20 Hz stimulation, *n* = 8; Fig. 4*H*). Second, we tested for latency differences between the low-threshold and high-threshold afferent stimulation. In CC cells, the average latency at low-threshold stimulation (25 µA) was 0.13 ± 0.06 ms (*n* = 8). Similarly, for cells with higher thresholds (>100 µA), the average latency was below 0.2 ms (*n* = 30). Together, these data suggest that Si events from distal dorsal root stim are monosynaptic (Nakatsuka et al., 2000; Doyle and Andresen, 2001; Torsney and MacDermott, 2006). Interestingly, CC Si events showed strong amplitude depression between the first and last eEPSC at 20 Hz stimulation (69 ± 1.65%, *n* = 8; Fig. 4*I*). This suggests that even though low-threshold primary afferents can be triggered reliably, they are subject to modulation or to short-term depression.

### *Atoh1*-lineage spinal cord neurons respond to optogenetic activation of *Pv*-lineage sensory neurons

**Figure 5.**
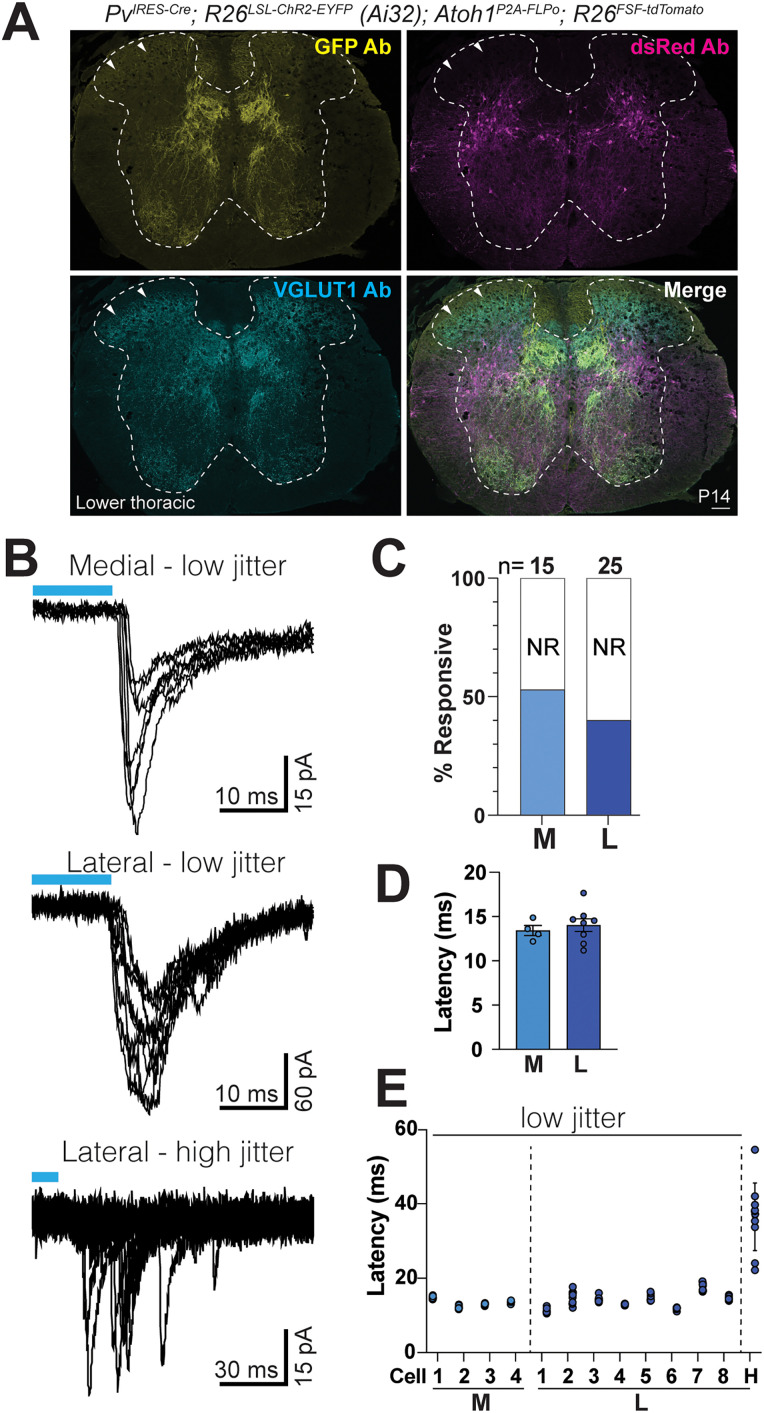
Optogenetic activation of *Pv*-lineage inputs onto *Atoh1*-lineage neurons. ***A***, ChR2 is expressed in *Pv*-lineage sensory neurons [*Pv^IRES-Cre^*; *R26^LSL-ChR2(H134R)-EYFP^* (Ai32)] and tdTomato in *Atoh1*-lineage spinal cord neurons (*Atoh1^P2A-FLPo^*; *R26^FSF-tdTom^*). VGLUT1 antibody labels afferent terminals of proprioceptive and low-threshold mechanoreceptors (LTMRs). *Pv*-lineage neurons expressing ChR2-EYFP (GFP Ab) have very few axons labeled in the LTMR recipient zone (arrowheads) indicating restricted expression to proprioceptors at P14. ***B***, Representative traces of a medial (7 sweeps) and lateral (10 sweeps) cell with low jitter and of a lateral cell with high jitter (10 sweeps). ChR2 is activated with blue light for ∼12 ms, the limit of the shutter speed of the X-Cite lamp. ***C***, The percentage of responsive M and L cells. ***D***, Apparent latencies for responsive M and L cells. ***E***, Apparent latencies of each M and L cell that responded to optogenetic stimulation (7 sweeps for M, 10 sweeps for L). Only one L cell had high jitter (H). Scale bar: 100 µm (***A***).

To determine whether *Atoh1*-lineage neurons receive PV^+^ sensory afferent information, we used a dual-recombinase strategy to express channelrhodopsin (ChR2) in *Pv*-lineage sensory afferents (*Pv^IRES-Cre^*; *R26^LSL-ChR2(H134R)-EYFP^*) and tdTomato in *Atoh1*-lineage cells (*Atoh1^P2A-FLPo^*; *R26^FSF-tdTom^*; Fig. 5*A*). *Pv^IRES-Cre^* mice are reported to label mostly proprioceptive afferents with <10% of low-threshold mechanoreceptors (LTMRs) labeled (de Nooij et al., 2013). Consistent with this, we find that *Pv^IRES-Cre^* labels sensory afferents with a proprioceptive innervation pattern and whose terminals express *vesicular glutamate transporter 1* (VGLUT1). Importantly, at age P14, we find that the LTMR recipient zone (Fig. 5*A*, arrowheads, VGLUT1^+^) has very few or no colocalization with *Pv^IRES-Cre^*-labeled sensory afferents indicating that few LTMRs are labeled at this age. In addition, PV is expressed in the adult spinal cord, but very few spinal cord neurons express PV prior to P15 (Fu et al., 2012; Yuengert et al., 2015). Therefore, all our recordings were performed from aged P10 to P14 mice. Upon blue light stimulation, we found optogenetically evoked EPSCs (oEPSCs; Fig. 5*B*). 53% medial (*n* = 15) and 36% lateral (*n* = 25) *Atoh1*-lineage cells responded to optogenetic stimulation of *Pv*-lineage labeled afferents (Fig. 5*C*). The considerable number of medial and lateral cells responding to optogenetic stimulation may reflect either axosomatic contacts, which are detectable by immunohistochemistry (Fig. 1*A’’,B’’,B’’’*), or axodendritic contacts with neurons whose soma are farther away from the densely innervated areas of *Pv*-lineage afferent axons. Approximately half (47%) of oEPSC-responsive cells showed events with more than one oEPSC, reminiscent of the multiple eEPSCs seen upon electrical stimulation. The following analyses pertain only to the first oEPSC. The apparent first latency (range, 11–18 ms) is comparable to what we previously reported for medial and lateral *Atoh1*-lineage neurons (Yuengert et al., 2015). Apparent first latencies were as follows: medial, 13.5 ± 0.57 ms, *n* = 4, and lateral, 14.0 ± 0.72 ms, *n* = 8, average ± SEM (Fig. 5*D*). Each individual cell's oEPSC latencies had low variability (Fig. 5*E*). Jitter was derived from the standard deviation of the latencies for each responsive cell. Jitter was as follows: medial, 0.35 ± 0.03 ms, *n* = 4, and lateral, 0.74 ± 0.17 ms, *n* = 8, average ± SEM. This low jitter suggests that the initial oEPSC events are monosynaptic. One lateral cell had a high jitter of 36.49 ± 6.46 ms, indicating that this cell received polysynaptic inputs (Fig. 5*E*).

### Presynaptic inputs to *Atoh1*-lineage neurons

**Figure 6.**
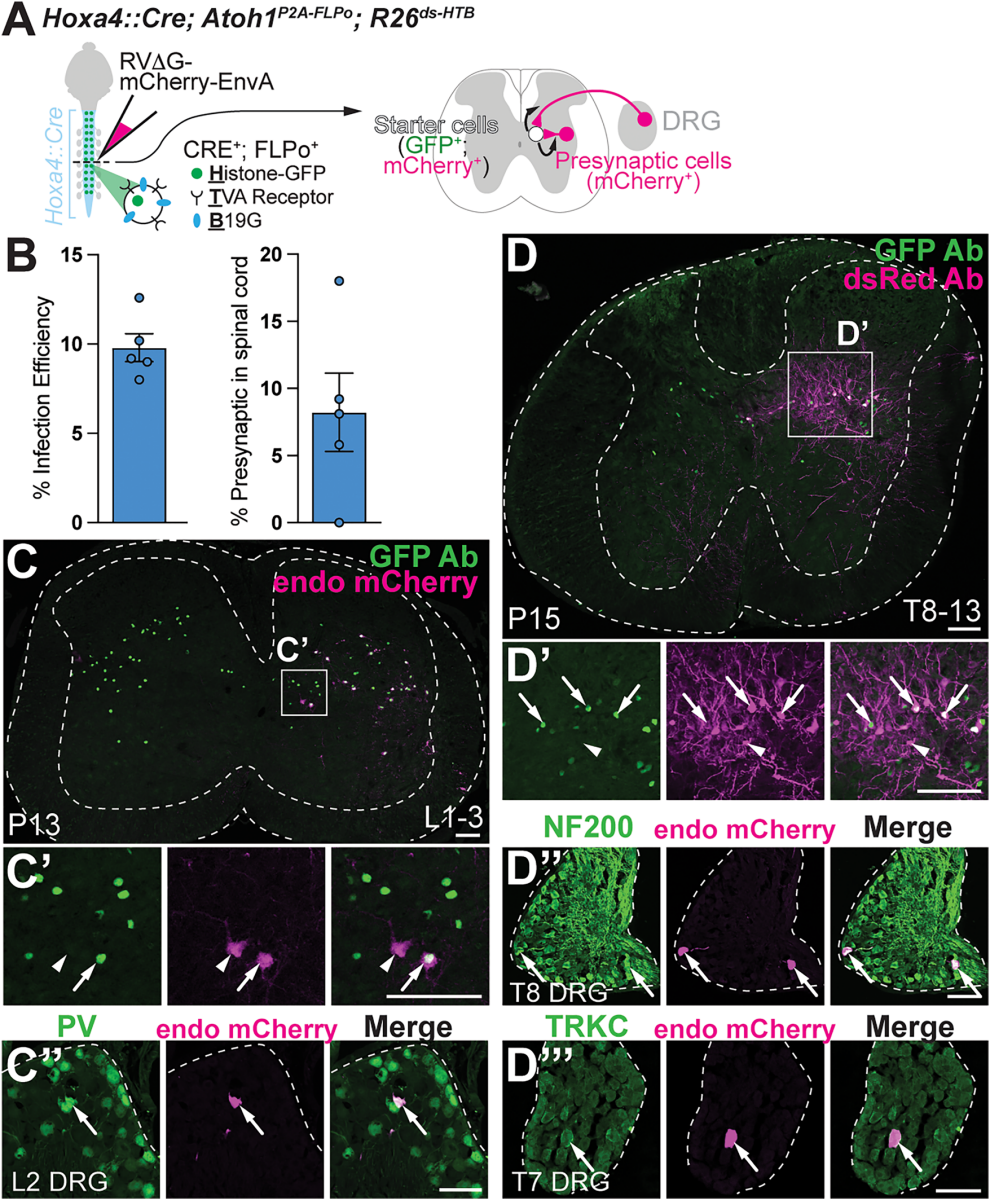
Diverse presynaptic inputs to *Atoh1*-lineage neurons. ***A***, Diagram of the monosynaptic rabies virus approach used to identify cells presynaptic to thoracolumbar *Atoh1*-lineage neurons. Cells containing both CRE and FLPo recombinase express the HTB allele (histone-GFP, TVA receptor, and B19G glycoprotein). Starter cells expressing the HTB allele and infected with RVΔG-mCherry-EnvA are GFP^+^ mCherry^+^. Presynaptic cells in the spinal cord and dorsal root ganglion (DRG) are mCherry^+^. ***B***, Infection efficiency at the injection site (GFP^+^mCherry^+^/total GFP^+^) and percentage of presynaptic cells in the spinal cord (mCherry^+^-only/total mCherry^+^) are shown. ***C–D’’’***, Two examples of unilateral spinal cord rabies virus injections. ***C,C’***, ***D,D’***, *Atoh1*-lineage starter cells (arrows) and local spinal cord presynaptic cells (arrowheads) can be seen at the site of injection. ***C’’***, An example of an mCherry^+^ cell in the DRG that expresses PV (arrow). ***D’’***, ***D’’’***, An example of two mCherry^+^ cells in the DRG that are myelinated (NF200^+^, arrows). An example of an mCherry^+^ cell in the DRG that expresses TRKC (arrow). All panels show endogenous mCherry fluorescence except for panels ***D*** and ***D’***, which shows amplification of the mCherry signal with dsRed antibody. Scale bars: 100 µm (***C–C’’***, ***D–D’’’***).

Optogenetic stimulation of *Pv*-lineage sensory afferents suggests that *Atoh1*-lineage neurons likely receive proprioceptive inputs (Fig. 5). As an orthogonal method to identify presynaptic inputs to *Atoh1*-lineage neurons, we used a retrograde monosynaptic G-deleted (ΔG) rabies virus tracing strategy (Wickersham et al., 2007a,b). This technology uses a complementation strategy to identify neurons presynaptic to the neurons of interest (Fig. 6*A*; Bourane et al., 2015; Callaway and Luo, 2015). We unilaterally injected rabies virus ΔG mCherry-EnvA into the thoracolumbar spinal cord of *Hoxa4::Cre; Atoh1^P2A-FLPo^; R26^ds-HTB^* mice. *Atoh1*-lineage starter cells express a nuclear histone-GFP and mCherry while presynaptic cells are mCherry-only (Fig. 6*C–D’’’*). We obtained low infection efficiency at the injection site (GFP^+^mCherry^+^/total GFP^+^, 9.8% ± 0.8% ms, *n* = 5) and very few presynaptic cells in the spinal cord (mCherry^+^-only/total mCherry^+^, 8.2% ± 2.9% ms, *n* = 5; Fig. 6*B*). Therefore, the data presented here are qualitative in nature. We find some presynaptic cells in the vicinity of *Atoh1*-lineage neurons in the intermediate spinal cord (Fig. 6*C,C’,D,D’*, arrowheads, *n* = 5 experiments, two representative experiments shown in *C* and *D*). No presynaptic cells were found in the superficial dorsal horn. Of the five injected spinal cords, only two injections had presynaptic mCherry^+^ cells in the DRG (Fig. 6*C’’,D’’,D’’’*). One experiment identified 2 mCherry^+^ DRG cells and the other identified 15 mCherry^+^ DRG cells, indicating a low incidence of labeling presynaptic DRG cells. These DRG mCherry^+^ cells were myelinated (NF200^+^, Fig. 6*D’’*) and expressed TRKC (Fig. 6*D’’’*) or PV (Fig. 6*C’’*), consistent with *Atoh1*-lineage neurons receiving large-diameter DRG neuron information.

## Discussion

Here, we characterize the passive and active membrane properties of neurons in the spinal cord that are reported to receive proprioceptive information and reside in laminae V–VII of the thoracolumbar spinal cord. In addition, we assess the organization of the synaptic inputs they receive from thoracic and lumbar dorsal roots. We report unique features of the CC and *Atoh1*-lineage populations.

### Clarke's column (CC)

CC neurons are a major contributor to the DSCT comprising 43–47% of spinocerebellar neurons projecting to the anterior zone of the cerebellum (Pop et al., 2022). Vast physiological literature has shown that DSCT neurons including CC cells are triggered by hindlimb muscle stretch and joint position (Oscarsson, 1965; Mann, 1973; Matsushita and Hosoya, 1979; Bosco and Poppele, 2001; Stecina et al., 2013). Anatomical studies show that CC axons are direct highways to granule cells in the cerebellum with very few if any detectable axon collaterals to the spinal cord, medulla, or cerebellar nuclei (Houchin et al., 1983; Walmsley and Nicol, 1990; Pop et al., 2022). Therefore, it is important to understand how this major cell type of the DSCT responds to electrical activity. Using the whole-cell patch-clamp technique, we found that they have a large capacitance consistent with their large morphology.

Although CC neurons are labeled with a genetic mouse line that is anatomically homogenous, we find that CC neurons are electrophysiologically heterogeneous. In addition, 56% of CC neurons are of the T-firing type, 32% of the F-firing type, and 12% of the *S*-firing type. Previous work also suggests that CC neurons are heterogeneous. Recordings in anesthetized or decerebrate cats found 34% of CC cells or 40% of DSCT cells fire spontaneously in vivo (Zytnicki et al., 1995; Fedirchuk et al., 2013), similar to the 45% of CC neurons that fire spontaneously in our acute slice preparation. These data suggest that CC neurons in cats and mice may have similar intrinsic firing properties in both in vivo and in vitro preparations. Furthermore, the inputs to CC neurons are heterogeneous (Hantman and Jessell, 2010). Whether the heterogeneity of firing types correlates with their differential input connectivity will be an interesting area for future work.

We find that CC neurons of the T-firing type increase their firing frequency linearly with increasing current injection, consistent with the literature in cats that CC neurons are frequency encoders of muscle activity (Holmqvist et al., 1956). CC neurons are reported to fire > 500 Hz in cats and with instantaneous frequencies as high as 1 kHz with little adaptation (Holmqvist et al., 1956; Kuno and Miyahara, 1968; Eide et al., 1969). In mice, we tested the ability of CC neurons to follow dorsal root stimulation of inputs up to 20 Hz. With a 500 pA depolarizing current injection for 1 s, the highest frequency reached in CC cells was 109 Hz (30 Hz on average).

Furthermore, we find that CC neurons express a signature hyperpolarization-activated current (I_h_). I_h_, known as the pacemaker current, was present in 94% of recorded CC neurons. The I_h_ current is driven by a family of hyperpolarization-activated cyclic nucleotide–gated (HCN) ion channels (Pape and McCormick, 1989; Ludwig et al., 1998). These channels are important for setting the resting membrane potential and signal integration and serve a pacemaker role in different cell types (DiFrancesco and Ojeda, 1980; McCormick and Huguenard, 1992; Maccaferri et al., 1993; Pape, 1996; Bal and McCormick, 1997; Robinson and Siegelbaum, 2003; Chalif et al., 2022). Single-cell RNA sequencing of spinocerebellar neurons suggests that *Hcn1* and *Hcn3* mRNA are expressed in CC neurons (Baek et al., 2019). Correspondingly, we find *Hcn1* and *Hcn3* transcripts colocalize with genetically labeled CC neurons. The I_h_ current could explain why recordings of DSCT neurons in cats show a “long-lasting repetitive discharge” that continues after a stimulus (Holmqvist et al., 1956; Kuno and Miyahara, 1968; Eide et al., 1969). More studies are needed to determine the importance of I_h_ or other currents in the repetitive firing of CC cells. In addition, it will be important to investigate how CC cells respond under the influence of neuromodulators or under physiologically relevant conditions such as locomotor behavior.

Lastly, we find that CC neurons in mice receive inputs from both distal and local dorsal roots, consistent with established literature demonstrating that CC neurons relay hindlimb and some thoracic proprioception (Szentagothai and Albert, 1955; Holmqvist et al., 1956; Eccles et al., 1961; Hantman and Jessell, 2010). Eight CC cells with single EPSC inputs were tested with 20 Hz lumbar root electrical stimulation. Very few CC neurons had eEPSC failures suggesting that these connections are monosynaptic. Interestingly, for single inputs to CC neurons, the average eEPSC amplitude decreased by 69% between the first and last eEPSCs at 20 Hz stimulation for 1 s. The depression is not due to inhibitory currents because these experiments were performed with inhibitory current blockers. Future experiments are needed to determine whether this eEPSC depression is due to neuromodulators or short-term depression.

### *Atoh1*-lineage neurons

To the best of our knowledge, this is the first report of the electrical activity of *Atoh1*-lineage neurons. *Atoh1*-lineage neurons have classically been subdivided into a contralaterally projecting medial and ipsilaterally projecting lateral population (Bermingham et al., 2001; Wilson et al., 2008). However, recent anatomical studies challenge this view with at least a subset of *Atoh1*-lineage lateral cells projecting potentially both ipsi- and contralaterally (Kaneyama and Shirasaki, 2018; Pop et al., 2022). Without unique molecular markers to distinguish subsets of the *Atoh1*-lineage population, we set out to evaluate whether the medial and lateral cells had distinct electrophysiological signatures. While *Atoh1*-lineage medial and lateral neurons had some significant differences in capacitance and rheobase, for the most part, they were very similar. Strikingly, even the percentage of firing types in medial compared with lateral neurons was very similar with most being the F-firing type. In contrast to CC neurons, *Atoh1*-lineage neurons were much smaller cells as indicated by their capacitance and had less of the T-firing type. We found no cells with an I_h_ current.

We also defined the organization and type of inputs to *Atoh1*-lineage neurons. Histological evidence suggests that some *Atoh1*-lineage medial and lateral neurons receive direct proprioceptive axosomatic proprioceptive contacts (Zampieri et al., 2014; Yuengert et al., 2015; Pop et al., 2022). We find here that *Atoh1*-lineage neurons have multiple sources of inputs. They received mainly local sensory afferent inputs, with more neurons responding to T11–T13 electrical dorsal root stimulation rather than lumbar root stimulation. In addition, 53% of *Atoh1*-lineage medial neurons and 36% of lateral neurons receive optogenetically activated EPSC inputs from *Pv*-lineage sensory neurons, which are largely proprioceptive, with most of them responding with a single oEPSC similar to the Si-type eEPSC seen upon electrical stimulation. These inputs are most likely monosynaptic due to the low jitter (Fig. 5). This also suggests that Ba-type afferents triggered electrically are polysynaptic sensory inputs. Using transsynaptic rabies virus tracing, we also find examples of large-diameter sensory neurons (NF200^+^, TRKC^+^, or PV^+^) as well as local interneurons in the intermediate spinal cord that are presynaptic to *Atoh1*-lineage neurons (Fig. 6).

We report here the electrophysiological characteristics of CC and *Atoh1*-lineage neurons, serving as a foundation for understanding how these cells respond to electrical activity. Furthermore, we find that CC and *Atoh1*-lineage neurons represent distinct proprioceptive circuit modules based on their input and output connectivity (Pop et al., 2022). This information will be valuable for future experiments focused on how these neurons convert sensory signals and communicate this information to motor circuits.

## Synthesis

Reviewing Editor: Matthew Grubb, King's College London Decisions are customarily a result of the Reviewing Editor and the peer reviewers coming together and discussing their recommendations until a consensus is reached. When revisions are invited, a fact-based synthesis statement explaining their decision and outlining what is needed to prepare a revision will be listed below. The following reviewer(s) agreed to reveal their identity: George Mentis.

Both reviewers are very positive about the revised manuscript. Please address all of their remaining minor concerns.

Reviewer 2

The study has been strengthened with the additional data provided by the authors. It is rather unfortunate that the authors did not perform further experiments with smaller steps of current to verify whether the "fading" firing neurons is due to depolarisation block or a true physiological characteristic. Irrespective, this is a minor issue. I have no additional concerns.

Reviewer 3

In this manuscript, the authors studied the electrophysiological properties and afferent inputs of Clarke's column (CC) and Atoh1-lineage neurons, two types of neurons in the mouse thoracic spinal cord involved in proprioception and located closely in laminae V-VII. They report differences in intrinsic and active properties between these two groups, such as the presence of Ih neurons in CC neurons, but not in the Atoh1-lineage. Additionally, they present evidence indicating that most CC neurons receive inputs from both distal (lumbar) and local (thoracic) sensory afferents from dorsal roots, whereas only a small proportion of Atoh1-lineage neurons exhibit this connectivity. Instead, Atho1-lineage neurons were more responsive to local dorsal root stimulation. Finally, by using optogenetics and rabis virus tracing, they show that Atho1-lineage neurons receive proprioceptive PV+ sensory inputs.

The experiments are technically well-performed and provide interesting data about the properties of these neurons. Moreover, the authors made a great effort to include different experimental approaches to obtain more information about the sensory inputs that they receive (e.g, root stimulation, optogenetics, transsynaptic tracing with rabies virus).

Overall, the quality of the data makes it convincing and interesting for researchers studying identity and connectivity of neurons in the spinal cord.

Below is a list of suggestions that would help clarifying the rationale of some experiments and a series of minor points:

The authors could comment on the reasons for choosing the mouse lines that they used for labelling the different types of neurons for the electrophysiological characterisation and discuss their level of specificity.

While authors categorized Atho1-lineage neurons into medial and lateral, some cells seem to be situated near the delineated "boundary" shown Fig.1B'&C , which raises questions about whether they were included in either group for analysis. It would be interesting to know if these neurons were indeed included in the analysis and to include a more detailed explanation of the criteria employed to assign them to one or the other group in the acute spinal cord slices.

Fig.4A nicely depicts a substantial innervation of PV-Chr2+ fibres surrounding medial Atho1-lineage neurons, while suggesting a lower density of connections into lateral Atho1-lineage neurons. However, the percentage of responsive cells (Fig.4C) is similar in both groups upon light stimulation. The authors should comment on this result.

Fig.6A. From my interpretation, the authors employed the Rosa26Lox-stop-LoxHTB mouse line to express TVA receptor and the rabies glycoprotein G in Atho1-lineage cells, as described in Li et al., PNAS 2013, 110(22) PMID: 23671081. It would be more informative if the authors develop more in details the schematic representation of mouse lines and tracing strategy that they used. Additionally, it would be beneficial to clarify the advantage of including Hoxa4::Cre mice. Why not directly utilize Atho1-Cre (mouse line included in Materials and Methods)?

## References

[B1] Abelew TA, Miller MD, Cope TC, Nichols TR (2000) Local loss of proprioception results in disruption of interjoint coordination during locomotion in the cat. J Neurophysiol 84:2709–2714. 10.1152/jn.2000.84.5.2709 11068014

[B2] Akay T, Tourtellotte WG, Arber S, Jessell TM (2014) Degradation of mouse locomotor pattern in the absence of proprioceptive sensory feedback. Proc Natl Acad Sci U S A 111:16877–16882. 10.1073/pnas.1419045111 25389309 PMC4250167

[B3] Baek M, Menon V, Jessell TM, Hantman AW, Dasen JS (2019) Molecular logic of spinocerebellar tract neuron diversity and connectivity. Cell Rep 27:2620–2635.e4. 10.1016/j.celrep.2019.04.113 31141687 PMC6555431

[B4] Bal T, McCormick DA (1997) Synchronized oscillations in the inferior olive are controlled by the hyperpolarization-activated cation current I(h). J Neurophysiol 77:3145–3156. 10.1152/jn.1997.77.6.3145 9212264

[B5] Bermingham NA, Hassan BA, Wang VY, Fernandez M, Banfi S, Bellen HJ, Fritzsch B, Zoghbi HY (2001) Proprioceptor pathway development is dependent on Math1. Neuron 30:411–422. 10.1016/S0896-6273(01)00305-1 11395003

[B6] Bosco G, Poppele RE (2001) Proprioception from a spinocerebellar perspective. Physiol Rev 81:539–568. 10.1152/physrev.2001.81.2.539 11274339

[B7] Bourane S, Grossmann KS, Britz O, Dalet A, Del Barrio MG, Stam FJ, Garcia-Campmany L, Koch S, Goulding M (2015) Identification of a spinal circuit for light touch and fine motor control. Cell 160:503–515. 10.1016/j.cell.2015.01.011 25635458 PMC4431637

[B8] Callaway EM, Luo L (2015) Monosynaptic circuit tracing with glycoprotein-deleted rabies viruses. J Neurosci 35:8979–8985. 10.1523/JNEUROSCI.0409-15.2015 26085623 PMC4469731

[B9] Cebrian C, Asai N, D'Agati V, Costantini F (2014) The number of fetal nephron progenitor cells limits ureteric branching and adult nephron endowment. Cell Rep 7:127–137. 10.1016/j.celrep.2014.02.033 24656820 PMC4049224

[B10] Chalif JI, Martinez-Silva ML, Pagiazitis JG, Murray AJ, Mentis GZ (2022) Control of mammalian locomotion by ventral spinocerebellar tract neurons. Cell 185:328–344.e6. 10.1016/j.cell.2021.12.014 35063074 PMC8852337

[B11] Chesler AT, et al. (2016) The role of PIEZO2 in human mechanosensation. N Engl J Med 375:1355–1364. 10.1056/NEJMoa1602812 27653382 PMC5911918

[B12] de Nooij JC, Doobar S, Jessell TM (2013) Etv1 inactivation reveals proprioceptor subclasses that reflect the level of NT3 expression in muscle targets. Neuron 77:1055–1068. 10.1016/j.neuron.2013.01.015 23522042 PMC3763960

[B13] DiFrancesco D, Ojeda C (1980) Properties of the current if in the sino-atrial node of the rabbit compared with those of the current iK, in Purkinje fibres. J Physiol 308:353–367. 10.1113/jphysiol.1980.sp013475 6262501 PMC1274552

[B14] Doyle MW, Andresen MC (2001) Reliability of monosynaptic sensory transmission in brain stem neurons in vitro. J Neurophysiol 85:2213–2223. 10.1152/jn.2001.85.5.2213 11353036

[B15] Eccles JC, Oscarsson O, Willis WD (1961) Synaptic action of group I and II afferent fibres of muscle on the cells of the dorsal spinocerebellar tract. J Physiol 158:517–543. 10.1113/jphysiol.1961.sp006783 13889058 PMC1359981

[B16] Edgley SA, Jankowska E (1988) Information processed by dorsal horn spinocerebellar tract neurones in the cat. J Physiol 397:81–97. 10.1113/jphysiol.1988.sp016989 3411521 PMC1192113

[B17] Eide E, Fedina L, Jansen J, Lundberg A, Vyklicky L (1969) Properties of Clarke's column neurones. Acta Physiol Scand 77:125–144. 10.1111/j.1748-1716.1969.tb04558.x 5348345

[B18] Fedirchuk B, Stecina K, Kristensen KK, Zhang M, Meehan CF, Bennett DJ, Hultborn H (2013) Rhythmic activity of feline dorsal and ventral spinocerebellar tract neurons during fictive motor actions. J Neurophysiol 109:375–388. 10.1152/jn.00649.2012 23100134

[B19] Fu Y, Sengul G, Paxinos G, Watson C (2012) The spinal precerebellar nuclei: calcium binding proteins and gene expression profile in the mouse. Neurosci Lett 518:161–166. 10.1016/j.neulet.2012.05.002 22579822

[B20] Gasparini S, DiFrancesco D (1997) Action of the hyperpolarization-activated current (Ih) blocker ZD 7288 in hippocampal CA1 neurons. Pflugers Arch 435:99–106. 10.1007/s004240050488 9359908

[B21] Grace AA, Bunney BS (1984a) The control of firing pattern in nigral dopamine neurons: burst firing. J Neurosci 4:2877–2890. 10.1523/JNEUROSCI.04-11-02877.1984 6150071 PMC6564720

[B22] Grace AA, Bunney BS (1984b) The control of firing pattern in nigral dopamine neurons: single spike firing. J Neurosci 4:2866–2876. 10.1523/JNEUROSCI.04-11-02866.1984 6150070 PMC6564731

[B23] Grace AA, Bunney BS (1986) Induction of depolarization block in midbrain dopamine neurons by repeated administration of haloperidol: analysis using in vivo intracellular recording. J Pharmacol Exp Ther 238:1092–1100. 3746660

[B24] Hamill OP, Marty A, Neher E, Sakmann B, Sigworth FJ (1981) Improved patch-clamp techniques for high-resolution current recording from cells and cell-free membrane patches. Pflugers Arch 391:85–100. 10.1007/BF00656997 6270629

[B25] Hantman AW, Jessell TM (2010) Clarke's column neurons as the focus of a corticospinal corollary circuit. Nat Neurosci 13:1233–1239. 10.1038/nn.2637 20835249 PMC2947611

[B26] Hippenmeyer S, Vrieseling E, Sigrist M, Portmann T, Laengle C, Ladle DR, Arber S (2005) A developmental switch in the response of DRG neurons to ETS transcription factor signaling. PLoS Biol 3:e159. 10.1371/journal.pbio.0030159 15836427 PMC1084331

[B27] Holmqvist B, Lundberg A, Oscarsson O (1956) Functional organization of the dorsal spino-cerebellar tract in the cat. V. Further experiments on convergence of excitatory and inhibitory actions. Acta Physiol Scand 38:76–90. 10.1111/j.1748-1716.1957.tb00174.x 13394332

[B28] Houchin J, Maxwell DJ, Fyffe RE, Brown AG (1983) Light and electron microscopy of dorsal spinocerebellar tract neurones in the cat: an intracellular horseradish peroxidase study. Q J Exp Physiol 68:719–732. 10.1113/expphysiol.1983.sp002761 6647745

[B29] Huang WH, Tupal S, Huang TW, Ward CS, Neul JL, Klisch TJ, Gray PA, Zoghbi HY (2012) Atoh1 governs the migration of postmitotic neurons that shape respiratory effectiveness at birth and chemoresponsiveness in adulthood. Neuron 75:799–809. 10.1016/j.neuron.2012.06.027 22958821 PMC3464459

[B30] Hughes DI, Sikander S, Kinnon CM, Boyle KA, Watanabe M, Callister RJ, Graham BA (2012) Morphological, neurochemical and electrophysiological features of parvalbumin-expressing cells: a likely source of axo-axonic inputs in the mouse spinal dorsal horn. J Physiol 590:3927–3951. 10.1113/jphysiol.2012.235655 22674718 PMC3476641

[B31] Kaneyama T, Shirasaki R (2018) Post-crossing segment of dI1 commissural axons forms collateral branches to motor neurons in the developing spinal cord. J Comp Neurol 526:1943–1961. 10.1002/cne.24464 29752714

[B32] Kuno M, Miyahara JT (1968) Factors responsible for multiple discharge of neurons in Clarke’s column. J Neurophysiol 31:624–638. 10.1152/jn.1968.31.4.624 5709876

[B33] Kuno M, Munoz-Martinez EJ, Randic M (1973) Sensory inputs to neurones in Clarke's column from muscle, cutaneous and joint receptors. J Physiol 228:327–342. 10.1113/jphysiol.1973.sp010089 4687101 PMC1331300

[B34] Li Y, Stam FJ, Aimone JB, Goulding M, Callaway EM, Gage FH (2013) Molecular layer perforant path-associated cells contribute to feed-forward inhibition in the adult dentate gyrus. Proc Natl Acad Sci U S A 110:9106–9111. 10.1073/pnas.1306912110 23671081 PMC3670356

[B35] Ludwig A, Zong X, Jeglitsch M, Hofmann F, Biel M (1998) A family of hyperpolarization-activated mammalian cation channels. Nature 393:587–591. 10.1038/31255 9634236

[B36] Maccaferri G, Mangoni M, Lazzari A, DiFrancesco D (1993) Properties of the hyperpolarization-activated current in rat hippocampal CA1 pyramidal cells. J Neurophysiol 69:2129–2136. 10.1152/jn.1993.69.6.2129 7688802

[B37] Madisen L, et al. (2010) A robust and high-throughput Cre reporting and characterization system for the whole mouse brain. Nat Neurosci 13:133–140. 10.1038/nn.2467 20023653 PMC2840225

[B38] Madisen L, et al. (2012) A toolbox of Cre-dependent optogenetic transgenic mice for light-induced activation and silencing. Nat Neurosci 15:793–802. 10.1038/nn.3078 22446880 PMC3337962

[B39] Madisen L, et al. (2015) Transgenic mice for intersectional targeting of neural sensors and effectors with high specificity and performance. Neuron 85:942–958. 10.1016/j.neuron.2015.02.022 25741722 PMC4365051

[B40] Mann MD (1971) Axons of dorsal spinocerebellar tract which respond to activity in cutaneous receptors. J Neurophysiol 34:1035–1050. 10.1152/jn.1971.34.6.1035 4329962

[B41] Mann MD (1973) Clarke's column and the dorsal spinocerebellar tract: a review. Brain Behav Evol 7:34–83. 10.1159/000124397 4349416

[B42] Matsushita M, Hosoya Y (1979) Cells of origin of the spinocerebellar tract in the rat, studied with the method of retrograde transport of horseradish peroxidase. Brain Res 173:185–200. 10.1016/0006-8993(79)90620-6 90539

[B43] McCormick DA, Huguenard JR (1992) A model of the electrophysiological properties of thalamocortical relay neurons. J Neurophysiol 68:1384–1400. 10.1152/jn.1992.68.4.1384 1331356

[B44] Miyoshi G, Hjerling-Leffler J, Karayannis T, Sousa VH, Butt SJ, Battiste J, Johnson JE, Machold RP, Fishell G (2010) Genetic fate mapping reveals that the caudal ganglionic eminence produces a large and diverse population of superficial cortical interneurons. J Neurosci 30:1582–1594. 10.1523/JNEUROSCI.4515-09.2010 20130169 PMC2826846

[B45] Nakagawa T, Yasaka T, Nakashima N, Takeya M, Oshita K, Tsuda M, Yamaura K, Takano M (2020) Expression of the pacemaker channel HCN4 in excitatory interneurons in the dorsal horn of the murine spinal cord. Mol Brain 13:127. 10.1186/s13041-020-00666-6 32948209 PMC7501643

[B46] Nakatsuka T, Ataka T, Kumamoto E, Tamaki T, Yoshimura M (2000) Alteration in synaptic inputs through C-afferent fibers to substantia gelatinosa neurons of the rat spinal dorsal horn during postnatal development. Neuroscience 99:549–556. 10.1016/S0306-4522(00)00224-4 11029546

[B47] Ogujiofor OW, Pop IV, Espinosa F, Durodoye RO, Viacheslavov ML, Jarvis R, Landy MA, Gurumurthy CB, Lai HC (2021) An Atoh1 CRE knock-in mouse labels motor neurons involved in fine motor control. eNeuro 8:ENEURO.0221-20.2021. 10.1523/ENEURO.0221-20.2021 PMC7901153 33468540

[B48] Osborn CE, Poppele RE (1988) The extent of polysynaptic responses in the dorsal spinocerebellar tract to stimulation of group I afferent fibers in gastrocnemius-soleus. J Neurosci 8:316–319. 10.1523/JNEUROSCI.08-01-00316.1988 3339414 PMC6569368

[B49] Oscarsson O (1965) Functional organization of the spino- and cuneocerebellar tracts. Physiol Rev 45:495–522. 10.1152/physrev.1965.45.3.495 14337566

[B50] Pape HC (1996) Queer current and pacemaker: the hyperpolarization-activated cation current in neurons. Annu Rev Physiol 58:299–327. 10.1146/annurev.ph.58.030196.001503 8815797

[B51] Pape HC, McCormick DA (1989) Noradrenaline and serotonin selectively modulate thalamic burst firing by enhancing a hyperpolarization-activated cation current. Nature 340:715–718. 10.1038/340715a0 2475782

[B52] Pop IV, et al. (2022) Structure of long-range direct and indirect spinocerebellar pathways as well as local spinal circuits mediating proprioception. J Neurosci 42:581–600. 10.1523/JNEUROSCI.2157-20.2021 34857649 PMC8805613

[B53] Qian K, Yu N, Tucker KR, Levitan ES, Canavier CC (2014) Mathematical analysis of depolarization block mediated by slow inactivation of fast sodium channels in midbrain dopamine neurons. J Neurophysiol 112:2779–2790. 10.1152/jn.00578.2014 25185810 PMC4254877

[B54] Richards CD, Shiroyama T, Kitai ST (1997) Electrophysiological and immunocytochemical characterization of GABA and dopamine neurons in the substantia nigra of the rat. Neuroscience 80:545–557. 10.1016/S0306-4522(97)00093-6 9284356

[B55] Robinson RB, Siegelbaum SA (2003) Hyperpolarization-activated cation currents: from molecules to physiological function. Annu Rev Physiol 65:453–480. 10.1146/annurev.physiol.65.092101.142734 12471170

[B56] Schindelin J, et al. (2012) Fiji: an open-source platform for biological-image analysis. Nat Methods 9:676–682. 10.1038/nmeth.2019 22743772 PMC3855844

[B57] Sherrington CS (1906) The integrative action of the nervous system. New Haven, CT: Yale University Press.

[B58] Stam FJ, Hendricks TJ, Zhang J, Geiman EJ, Francius C, Labosky PA, Clotman F, Goulding M (2012) Renshaw cell interneuron specialization is controlled by a temporally restricted transcription factor program. Development 139:179–190. 10.1242/dev.071134 22115757 PMC3231776

[B59] Stecina K, Fedirchuk B, Hultborn H (2013) Information to cerebellum on spinal motor networks mediated by the dorsal spinocerebellar tract. J Physiol 591:5433–5443. 10.1113/jphysiol.2012.249110 23613538 PMC3853486

[B60] Szentagothai J, Albert A (1955) The synaptology of Clarke's column. Acta Morphol Acad Sci Hung 5:43–51. 14375853

[B61] Torsney C, MacDermott AB (2006) Disinhibition opens the gate to pathological pain signaling in superficial neurokinin 1 receptor-expressing neurons in rat spinal cord. J Neurosci 26:1833–1843. 10.1523/JNEUROSCI.4584-05.2006 16467532 PMC6793628

[B62] Tu H, Deng L, Sun Q, Yao L, Han JS, Wan Y (2004) Hyperpolarization-activated, cyclic nucleotide-gated cation channels: roles in the differential electrophysiological properties of rat primary afferent neurons. J Neurosci Res 76:713–722. 10.1002/jnr.20109 15139030

[B63] Tuthill JC, Azim E (2018) Proprioception. Curr Biol 28:R194–R203. 10.1016/j.cub.2018.01.064 29510103

[B64] Walmsley B, Nicol MJ (1990) Location and morphology of dorsal spinocerebellar tract neurons that receive monosynaptic afferent input from ankle extensor muscles in cat hindlimb. J Neurophysiol 63:286–293. 10.1152/jn.1990.63.2.286 2313345

[B65] Wickersham IR, Finke S, Conzelmann KK, Callaway EM (2007a) Retrograde neuronal tracing with a deletion-mutant rabies virus. Nat Methods 4:47–49. 10.1038/nmeth999 17179932 PMC2755236

[B66] Wickersham IR, Lyon DC, Barnard RJ, Mori T, Finke S, Conzelmann KK, Young JA, Callaway EM (2007b) Monosynaptic restriction of transsynaptic tracing from single, genetically targeted neurons. Neuron 53:639–647. 10.1016/j.neuron.2007.01.033 17329205 PMC2629495

[B67] Wilson SI, Shafer B, Lee KJ, Dodd J (2008) A molecular program for contralateral trajectory: Rig-1 control by LIM homeodomain transcription factors. Neuron 59:413–424. 10.1016/j.neuron.2008.07.020 18701067

[B68] Yang H, Xie X, Deng M, Chen X, Gan L (2010) Generation and characterization of Atoh1-Cre knock-in mouse line. Genesis 48:407–413. 10.1002/dvg.20633 20533400 PMC2885570

[B69] Yuengert R, Hori K, Kibodeaux EE, McClellan JX, Morales JE, Huang TP, Neul JL, Lai HC (2015) Origin of a non-Clarke's column division of the dorsal spinocerebellar tract and the role of caudal proprioceptive neurons in motor function. Cell Rep 13:1258–1271. 10.1016/j.celrep.2015.09.064 26527010 PMC4644487

[B70] Zampieri N, Jessell TM, Murray AJ (2014) Mapping sensory circuits by anterograde transsynaptic transfer of recombinant rabies virus. Neuron 81:766–778. 10.1016/j.neuron.2013.12.033 24486087 PMC3988472

[B71] Zhang ZW (2003) Serotonin induces tonic firing in layer V pyramidal neurons of rat prefrontal cortex during postnatal development. J Neurosci 23:3373–3384. 10.1523/JNEUROSCI.23-08-03373.2003 12716945 PMC6742316

[B72] Zytnicki D, Lafleur J, Kouchtir N, Perrier JF (1995) Heterogeneity of contraction-induced effects in neurons of the cat dorsal spinocerebellar tract. J Physiol 487:761–772. 10.1113/jphysiol.1995.sp020916 8544137 PMC1156661

